# Characterization of the transcriptome of *Haloferax volcanii*, grown under four different conditions, with mixed RNA-Seq

**DOI:** 10.1371/journal.pone.0215986

**Published:** 2019-04-30

**Authors:** Sebastian Laass, Vivian A. Monzon, Jana Kliemt, Matthias Hammelmann, Friedhelm Pfeiffer, Konrad U. Förstner, Jörg Soppa

**Affiliations:** 1 Goethe University, Biocentre, Frankfurt, Germany; 2 University of Würzburg, Core Unit Systems Medicine, Institute for Molecular Infection Biology (IMIB), Würzburg, Germany; 3 Computational Biology Group, Max-Planck-Institute of Biochemistry, Martinsried, Germany; Keio University, JAPAN

## Abstract

*Haloferax volcanii* is a well-established model species for haloarchaea. Small scale RNomics and bioinformatics predictions were used to identify small non-coding RNAs (sRNAs), and deletion mutants revealed that sRNAs have important regulatory functions. A recent dRNA-Seq study was used to characterize the primary transcriptome. Unexpectedly, it was revealed that, under optimal conditions, *H*. *volcanii* contains more non-coding sRNAs than protein-encoding mRNAs. However, the dRNA-Seq approach did not contain any length information. Therefore, a mixed RNA-Seq approach was used to determine transcript length and to identify additional transcripts, which are not present under optimal conditions. In total, 50 million paired end reads of 150 nt length were obtained. 1861 protein-coding RNAs (cdRNAs) were detected, which encoded 3092 proteins. This nearly doubled the coverage of cdRNAs, compared to the previous dRNA-Seq study. About 2/3 of the cdRNAs were monocistronic, and 1/3 covered more than one gene. In addition, 1635 non-coding sRNAs were identified. The highest fraction of non-coding RNAs were cis antisense RNAs (asRNAs). Analysis of the length distribution revealed that sRNAs have a median length of about 150 nt. Based on the RNA-Seq and dRNA-Seq results, genes were chosen to exemplify characteristics of the *H*. *volcanii* transcriptome by Northern blot analyses, e.g. 1) the transcript patterns of gene clusters can be straightforward, but also very complex, 2) many transcripts differ in expression level under the four analyzed conditions, 3) some genes are transcribed into RNA isoforms of different length, which can be differentially regulated, 4) transcripts with very long 5’-UTRs and with very long 3’-UTRs exist, and 5) about 30% of all cdRNAs have overlapping 3’-ends, which indicates, together with the asRNAs, that *H*. *volcanii* makes ample use of sense-antisense interactions. Taken together, this RNA-Seq study, together with a previous dRNA-Seq study, enabled an unprecedented view on the *H*. *volcanii* transcriptome.

## Introduction

Small non-coding RNAs (sRNAs) exist in all three domains of life, archaea, bacteria, and eukaryotes. They were found to fulfill important regulatory roles and are involved in many biological functions, e.g. stress adaptation, metabolic regulation, and pathogenesis. In eukaryotes, altered expression levels of so called microRNAs (miRNAs), very small RNAs of about 22 nt, are associated with diseases. Several recent reviews summarize the current knowledge about sRNAs in eukaryotes [1–3], in bacteria [4–11], and in archaea [11–14].

The first archaeal sRNAs were discovered more than 10 years ago in *Archaeoglobus fulgidus* [15] and in *Sulfolobus solfataricus* [16]. A few years later, sRNAs were also detected in *Haloferax volcanii* [17]. The numbers of sRNAs that were detected in these early studies were rather limited. However, in recent years RNA-Seq or dRNA-Seq studies have been performed with several archaeal species that gave overviews of the sRNA repertoires of *Haloferax volcanii* [18, 19], *Methanolobus psychrophilus* [20], *Thermococcus kodakarensis* [21], *Pyrococcus abyssi* [22], *Sulfolobus solfataricus* [23], and *Methanosarcina mazei* Gö1 [24]. S1 Table summarizes the numbers of annotated protein-coding genes as well as the numbers of experimentally detected protein-coding mRNAs and non-coding sRNA of these species. The numbers of sRNAs varied widely, both for intergenic sRNAs (igRNAs) and especially for cis-antisense sRNAs (asRNAs). For example, *M*. *mazei* contained only 199 igRNAs and 43 asRNAs, while 395 igRNAs and 1244 asRNAs were found for *H*. *volcanii*. In contrast, the numbers of protein-coding genes were not very different for these species (3371 versus 4040).

Application of differential RNA-Seq (dRNA-Seq) [25] ensures that primary transcripts rather than processing products are analyzed. Characterization of the primary transcriptome of *H*. *volcanii* with dRNA-Seq led to the discovery of a very large number of nearly 2800 novel sRNAs [19]. Due to application of dRNA-Seq, these sRNAs represent primary transcripts. However, the dRNA-Seq approach concentrates solely on the 5’-end of transcripts (with a read length of about 150 nt). It does not contain any information about transcript lengths, because the protocol does not contain a fragmentation step of the RNAs (compare Fig 1 in [25]). To overcome this limitation, and to elucidate transcript lengths, an RNA-Seq approach was chosen in the present study. It should be noted, that RNA-Seq cannot discriminate between primary and processed transcripts. In addition, when the total numbers of reads are identical, the sequencing depth is lower for RNA-Seq than for dRNA-Seq, because the reads are distributed over the whole transcript length, and are not concentrated at the 5’-ends alone. Therefore, the RNA-Seq and dRNA-Seq data sets are complementary to each other and are an excellent combination to deepen the overview of the *H*. *volcanii* transcriptome.

**Table 1:** Culture conditions for mixed RNA-Seq.

No.	Medium	Growth phase	NaCl conc.	Abbreviation (mnemomic)
1	Complex	Exponential	2.2 M	E (exponential)
2	Complex	Stationary	2.2 M	S (stationary)
3	Complex	Exponential	0.9 M	L (low salt)
4	Synthetic with glucose	Exponential	2.2 M	G (glucose)

The dRNA-Seq study had been performed with cultures that had been grown in complex medium under optimal conditions to mid-exponential growth phase. Transcripts were found for less than half of the protein-coding genes. This could be expected, because many functions are not needed under these conditions, e.g. enzymes for amino acid and other syntheses, proteins involved in chemotaxis or biofilm formation, and proteins involved in stress responses. To broaden the view, and enable the detection of further, differentially regulated protein-coding transcripts, this study analyzed cultures grown under four different conditions. Equal fractions of RNAs from the four cultures were mixed and the resulting RNA-pool was used for library preparation using one set of primers and was sequenced using RNA-Seq. A similar approach has previously been performed with *Paenibacillus riograndensis* by the Wendisch group. A pool of RNAs from cultures grown under 15 different conditions were used [26]. The “mixed RNA-Seq” approach is very cost-effective and informative. However, because average signals from several conditions (four in this study) are generated, subsequent analyses are needed for the detailed characterization of individual genes. Here, we applied Northern Blot analyses, and 14 probes were used to exemplify the strength of “mixed RNA-Seq” as well as characteristic features of the transcriptome of *H*. *volcanii*.

## Results and discussion

### Characterization of the *H*. *volcanii* transcriptome using mixed RNA-Seq

Recently we have analyzed the primary transcriptome of *H*. *volcanii* cultures growing under optimal conditions using dRNA-Seq [19]. In total, 4749 transcription start sites (TSS) were discovered. Remarkably, the number of non-coding RNAs (2792) was considerably higher than the number of protein-coding RNAs (1851). Due to the experimental approach of dRNA-Seq, it was confirmed that all these 2792 non-coding RNAs were primary transcripts. However, the results did not contain any length information. Therefore, as a follow-up, an RNA-Seq study was performed with the major goal to analyze the length distribution of *H*. *volcanii* transcripts.

In addition, under optimal conditions transcripts of only 1851 of the about 4000 annotated protein-coding genes had been detected in the dRNA-Seq study. It can be expected that further genes are transcribed under non-optimal conditions. Therefore, RNA-Seq was not confined to optimal conditions, but three additional conditions were chosen to increase the width of representation of the transcriptome. Non-standard conditions were chosen to deviate strongly, anticipating that many additional genes would be expressed. The four conditions included growing as well as stationary phase cultures, cultures grown in synthetic medium and under low salt stress (Table 1). Total RNA was isolated from cultures grown under the four conditions. The total absence of contaminating DNA was ensured using a bioanalyzer and, moreover, by PCR analyses in the absence of a reverse transcription step. Equal amounts of the four RNA preparations were mixed and the RNA-Seq analysis was performed on this mixed probe. This approach of “mixed RNA-Seq” has the advantage of being very cost-effective, i.e. only one instead of four library preparations is required. Of course, the disadvantage is that average signals are generated and downstream analyses are required to characterize the transcripts that are present under the four individual conditions. RNA-Seq was performed by the company StarSeq (Mainz, Germany, www.starseq.com) using standard procedures. About 50 million strand-specific paired end reads of 150 nt were generated (24.7 million read 1, 26.2 million read 2). The results are available at the GEO database (www.ncbi.nlm.nih.gov/geo/query/acc.cgi?acc=GSE119686). All reads could be mapped to the genome of *H*. *volcanii*, and 43 million reads were properly paired. The removal of rRNA (performed by StarSeq) had been extremely efficient, because only 45 906 reads mapped to the 16S and 23S rRNA genes. The software Annogesic [27] was used to predict the presence and length of transcripts from the RNA-Seq results. The parameters of transcript detection were optimized to increase true positives and simultaneously reduce false positives. For example, minimal average read counts from 5 to 105 were used for gene prediction, and the results were estimated by visual inspection in a genome browser. It should be noted that a metaanalysis of six different methods revealed that visual inspection after bioinformatics analysis is very powerful and in fact better than several different bioinformatics analysis methods alone [28]. An average read count of 70 was chosen as a lower limit for the prediction of transcripts, because it combined a high prediction rate and a low rate of obvious false positive predictions. On the major chromosome, 2833 transcripts of four different classes were detected with the limit of 70. The number of predicted transcripts was not very sensitive to the read count setting, e.g. a lower limit of 80 led to the prediction of 2755 transcripts, and a lower limit of 60 led to the prediction of 3006 transcripts. Of course, considerably lower limits led to very high numbers, e.g. 5657 “transcripts” were predicted with a limit of 10. However, most or all of the additional transcripts turned out to be false positives upon visual inspection.

Often the reads were evenly distributed over the whole length of annotated ORFs, allowing an easy and unambiguous transcript prediction. One example is shown in S1A Fig. However, also large variations of read counts over the length of ORFs were observed, and an example for an especially uneven distribution is shown in S1B Fig. This fits the expectation, because it is well-known that sequence sampling is not uniform throughout the transcriptome [29]. Most regions of the genome were well transcribed. S1C Fig shows a region where the genes are evenly distributed on both DNA strands, and transcripts for all genes were detected by RNA-Seq. In contrast, S1D Fig shows a genomic region that did not exhibit any transcription under the four analyzed culture conditions. However, this was very rare, and there were only about 10 examples where more than five consecutive genes were not transcribed. Two examples were genes for the synthesis of pilins and the operon for anaerobic nitrate respiration, most other examples were comprised of genes of unknown function. By far the largest non-transcribed region was comprised of 92 genes on replicon pHV4 (genes HVO_A0017 to HVO_A0108). More than half of the genes (48) were annotated as “conserved hypotheticals”, indicating that this region encodes (conserved) functions, which are not needed under any of the four tested conditions. According to phiSpy [30], the region from HVO_A0005 to A0062 is predicted to represent a prophage. Prophages typically code for large numbers of genes that lack a functional assignments.

Most transcripts were classified into the four classes of protein-coding transcripts (cdRNAs), cis antisense transcripts (asRNAs), internal sense transcripts (isRNAs), and intergenic transcripts (igRNAs) (S2 Table). The classification was based on visual inspection of the read distribution and the transcript prediction in the Integrated Genome Browser. In a few cases the predicted transcript was classified as a potential false positive upon visual inspection (no transcription start site in the dRNA-Seq results and very low read for considerably fractions of the ORF), and thus no class was assigned (empty cells in S2 Table). On the other hand, in very few cases the predicted RNA (> 70 reads) was much shorter than the annotated ORF, but a low sequence coverage was found over the whole ORF and the dRNA-Seq results showed the presence of a transcription start site. In these cases the bioinformatics transcript length prediction was not changed, but the transcript was nevertheless classified as cdRNA.

All RNA-Seq results are summarized in the S2 Table, including start and stop of the predicted transcripts, transcript lengths, transcript class, affiliated gene, and further information. Table 2 gives an overview of the numbers of transcripts of the four classes that were found to be expressed from the four chromosomes of *H*. *volcanii*. The largest class was cdRNAs, which constituted 53% of all transcripts. In the recent dRNA-Seq study [19] only 39% of all transcripts were found to be protein-coding, and thus the majority belonged to the three classes of non-coding RNAs. This “discrepancy” (at first sight) can be explained by the very different sequencing depths of the two studies. In the dRNA-Seq study 200 million reads were generated, that were concentrated on the 5’-ends of the transcripts. In the RNA-Seq study 50 million reads were generated, that were distributed over the whole length of the transcripts. It had already been observed in the dRNA-Seq study that many of the non-coding RNAs had low transcript levels (e.g. Fig 5 in [19]). Such low-abundance non-coding transcripts were below detection limit in the current RNA-Seq study. This explains the higher relative fraction of cdRNAs. In addition, characterization of the transcriptomes under three additional conditions preferentially increased the number of cdRNAs rather than that of non-coding RNAs (see below).

**Table 2:** Summary of predicted transcripts. The transcripts were classified as protein-coding transcripts (cdRNAs), cis antisense transcripts (asRNAs), internal sense transcripts (isRNAs), and intergenic transcripts (igRNAs). They are localized on the major chromosome (Chr) and the three minor chromosomes pHV1, pHV3, and pHV4 (the investigated strain is devoid of small plasmid pHV2).

RNA class	Chr.	pHV1	pHV3	pHV4	Sum	%
cdRNAs	1414	44	156	247	1861	53
asRNAs	986	49	39	148	1222	35
isRNAs	178	3	15	17	213	6
igRNAs	157	11	12	20	200	6
Sum	2735	107	222	432	3496	100

The second largest class was asRNAs, with 35% of all transcripts. Notably, the number of asRNAs was about sevenfold higher than the number of igRNAs, indicating that the sense-antisense interaction might potentially be a prominent mechanism of gene regulation in *H*. *volcanii*. It has been observed before that in *H*. *volcanii* the number of asRNAs is much higher than the number of igRNAs [18, 19]. Two observations indicate that many asRNAs might be negative regulators of their cognate mRNAs: 1) the levels of many asRNAs and mRNAs are anti-correlated [19], and 2) the level changes of various asRNAs and mRNAs after an oxidative stress were also anti-correlated [18]. Recently, asRNAs have been discovered in the transcriptomes of various species of prokaryotes, including *E*. *coli* and gut microbioty [31–33]. Nevertheless, the number of asRNAs in the *H*. *volcanii* transcriptome is exceptionally high.

A few further RNAs were detected, in addition to the RNAs of the four classes discussed above, which are included in Table 2. These include the stable RNAs involved in translation, e.g. 16S rRNA, 23S rRNA, and 29 tRNAs. In addition, RNase P and CRISPR were found. It should be noted that *H*. *volcanii* and other haloarchaea contain only very few modified nucleotides in tRNAs and in rRNAs, in stark contrast to other archaeal species [34]. It has been hypothesized that the high salt concentration in the cytoplasm enables folding of unmodified RNAs, which need nucleotide modifications for folding in mesohalic species [34]. Accordingly, the number of snoRNAs is very small, and only three CD box snoRNAs and two H/ACA snoRNAs (on one transcript) have been predicted to be present in *H*. *volcanii* [34, 35]. All four transcripts have been detected by mixed RNA-Seq.

As exemplified in the following paragraphs, the mixed RNA-Seq results can be used to derive hypotheses and design subsequent studies.

### Analysis of the coding capacity of “non-coding RNAs”

The RNA-Seq study had led to the discovery of 1635 transcripts, which did not match any annotated protein-coding gene and were thus categorized as “non-coding” (Table 2). The coding capacity of *H*. *volcanii* is well annotated [36]. This includes efforts to reduce missing gene calls by comparison to annotated genomes from more than a dozen genomes from the genus Haloferax [37, 38] and to haloarchaea with extensive proteomic validation, including an analysis tailored for the small proteome [39–42]. These efforts were extended by a BLASTx comparison of all intergenic regions to the UniProt database in 2016 [19]. Nevertheless, the possibility existed that a fraction of the “non-coding” RNAs might encode additional, typically very small, proteins that have escaped direct mass-spectrometric detection as yet, and that are not well conserved or are missing gene calls in other species. To test this possibility, all 1635 transcripts of the three non-coding RNA classes (Table 2) were translated in all three reading frames. ATG and GTG were used as possible start codons, which represent about 98% of the start codons of protein-coding genes of *H*. *volcanii*. Haloarchaea have a very acidic proteome and the fraction of proteins with an isoelectric point (pI) above 6.0 is very small [43]. In fact, 87% of the 4074 annotated proteins of *H*. *volcanii* have an pI of up to 6.0, and only 13% have an pI of higher than 6.0. Similarly, 76% of all annotated small proteins up up to 150 amino acids have a pI of lower than 6.0, and only 24% have a higher pI. Therefore, the results were restricted to proteins with an pI of up to pH 6.0, a restriction that should lead to only very few false negatives (overlooked true proteins). A further restriction was a minimum of 40 codons. The analysis yielded 121 ORFs matching the above-mentioned criteria. Haloarchaea have a very specific codon usage, therefore, the haloarchaeal codon usage table is of high predictive value for the identification of real protein-coding genes [44]. A *H*. *volcanii*-specific codon usage Table was computed from the more than 4000 protein coding genes of *H*. *volcanii* (S3 Table). A codon usage Table was also computed for the 1077 small proteins of up to 150 amino acids (S3 Tabble). As expected, the codon usages of all proteins and the small proteins are identical (99.96% correlation), because both groups rely on the same set of charged tRNAs. However, the haloarchaeal codon usage is very different from that of *E*. *coli* (major differences are colored in the S3 Table). The high usage of some codons (e.g. 92% GAC) and low usage of other codons (e.g. 1% UUA) makes the haloarchaeal codon usage table especially informative for the discrimination between real protein-coding genes and false positive open reading frames. The program “codonpreference” from the GCG package [45] was used to analyze which of the 121 ORFs exhibit the codon usage computed from the *Haloferax* protein-coding genes. None of the igRNAs, only a single asRNA, and two isRNAs contained an ORF which reflects the haloarchaeal codon usage.

The single asRNA with an ORF exhibiting the haloarchaeal codon usage was antisense to the annotated protein coding gene HVO_C0026, which encodes a non-conserved “hypothetical protein”. While the asRNA could be detected in the RNA-Seq as well as in the dRNA-Seq results, the mRNA *hvo_C0026* could not be detected at all. Therefore, it seems that the “asRNA” is a simple protein-coding mRNA (cdRNA) and the annotated ORF on the opposite strand does not code for a protein (spurious ORF).

Of course for the isRNAs one of the three frames is the frame of the annotated protein-coding gene and thus exhibits the haloarchaeal codon usage throughout the whole isRNA. The analysis was only taken as indicative for the presence of a putative novel protein when a rise and a decline of the codonpreference was observed within the isRNA, and this was found in only two cases. These two isRNAs were internal to HVO_1608 and HVO_1615, and thus the possibility exists that the annotation is too long, the actual genes are shorter and the RNAs belong to the group of cdRNAs. However, in both cases multiple sequence alignments of the proteins indicated that the annotation is correct, which would mean that the isRNAs are probable degradation intermediates and are not translated.

Taken together, the analysis of the coding potential revealed that none or at most extremely few of the 1635 non-coding RNAs were actually translated into haloarchaeal proteins. This underscored that *H*. *volcanii* contains an extremely high number of non-coding RNAs.

### Selection of examples for Northern blot analyses

An integrated analysis of genome annotation, the previously obtained dRNA-Seq results [19] and data from this RNA-Seq study yielded important information about the transcriptome of *H*. *volcanii*. Various observations were validated by Northern blot analyses. In order to discriminate between the four conditions under study, RNAs were isolated separately from cultures grown under each of the four conditions. All Northern blot experiments were performed as three independent biological replicates.

The following features were addressed and exemplified by the results of RNA-Seq, dRNA-Seq, and Northern blots: 1) length determination of cdRNAs and especially of the novel non-coding sRNAs, 2) elucidation of operon structures, 3) discrimination between very long 5’-UTRs and igRNAs upstream of coding regions, 4) the existence of very long 5’- and 3’-UTRs, 5) the fraction of genes with overlapping 3’-UTRs, and 6) characterization of differential expression of genes. These points are discussed in the following paragraphs.

### Length distributions of non-coding and of coding transcripts

**Fig 1:**
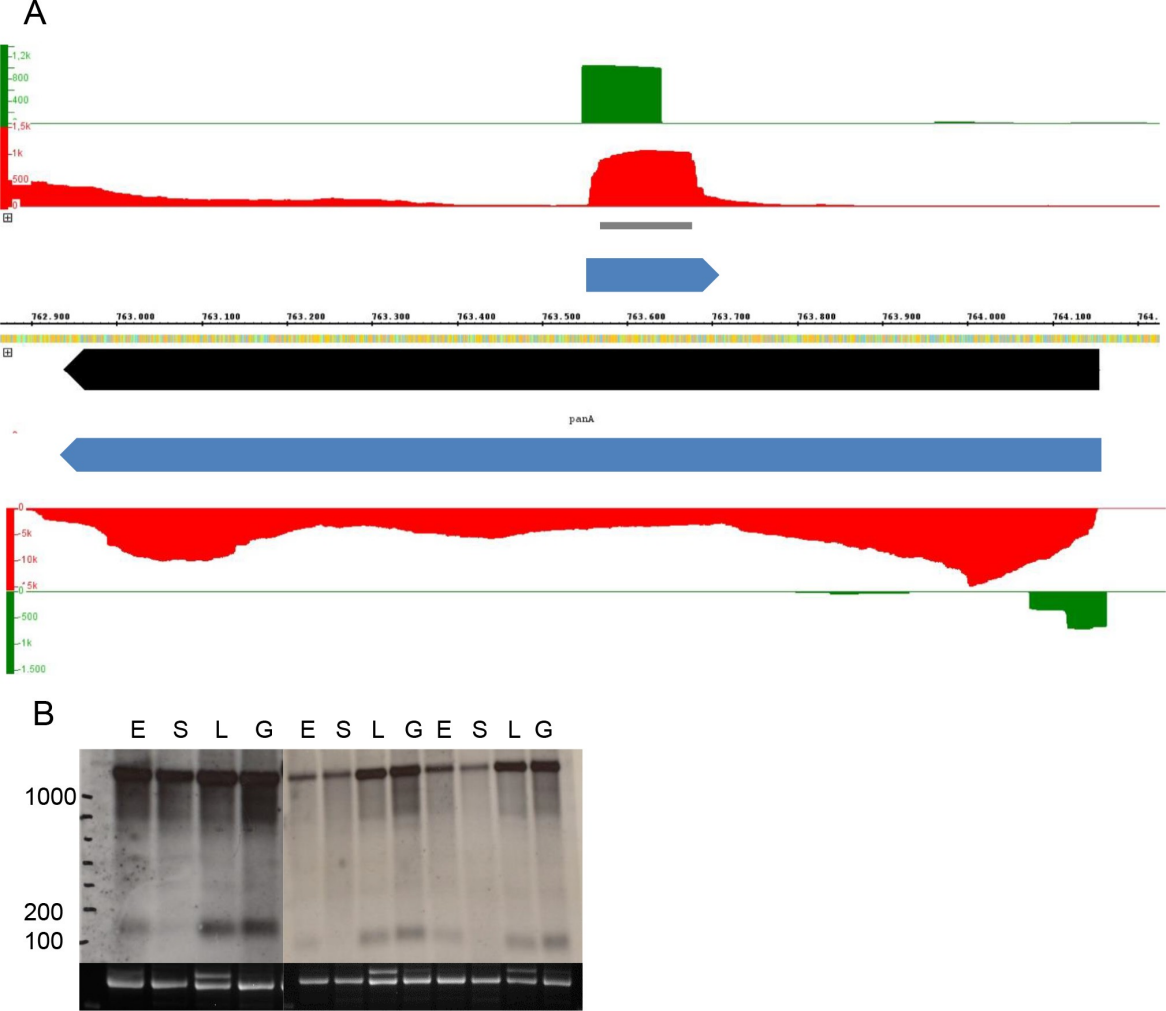
Determination of the length of a protein-coding cdRNA and its asRNA. **A.** Screenshot from the Integrated Genome Browser. The middle line represents the respective replicon and the numbers the genomic coordinated. The following data are shown in the panels from the replicon line to the top/bottom: gene annotations (black), transcript prediction based on Northern blot analysis (blue), RNA-Seq results (red), and dRNA-Seq results (green). The y-axis in the RNA-Seq/dRNA-Seq panels show the number of reads. The localization of the probe used for Northern blot analysis is indicated in grey. **B.** Northern blot analysis. The mnemomics of the four conditions are explained in Table 1. The results from three biological replicates are shown. Upper part: Hybridization signals. Lower part: 16S rRNA.

One important goal for this RNA-Seq study was the determination of the length distributions of novel non-coding RNAs, which had been discovered in the recent dRNA-Seq study [19]. One example is shown in Fig 1. The protein-coding gene *panA* is encoded on the bottom strand. A TSS had been detected (Fig 1A, green) and the RNA-Seq reads are distributed over the whole annotated ORF (Fig 1A, red). As typical for protein-encoding transcripts from *H*. *volcanii*, the transcript is leaderless and includes a 3’-UTR. The dRNA-Seq study led to the identification of a TSS for an asRNA on the top strand. The RNA-Seq data verified the existence of the asRNA and revealed that is had a length of about 150 nt. Both the asRNA and the cdRNA could also be observed in Northern blot analyses (Fig 1B), and the sizes detected by RNA-Seq and by Northern blots were in excellent agreement. The Northern blots also revealed that the asRNA is differentially regulated and has a much lower abundance in stationary phase than at the three other conditions. Differential expression for the four different conditions will be addressed in a separate paragraph (see below).

**Fig 2:**
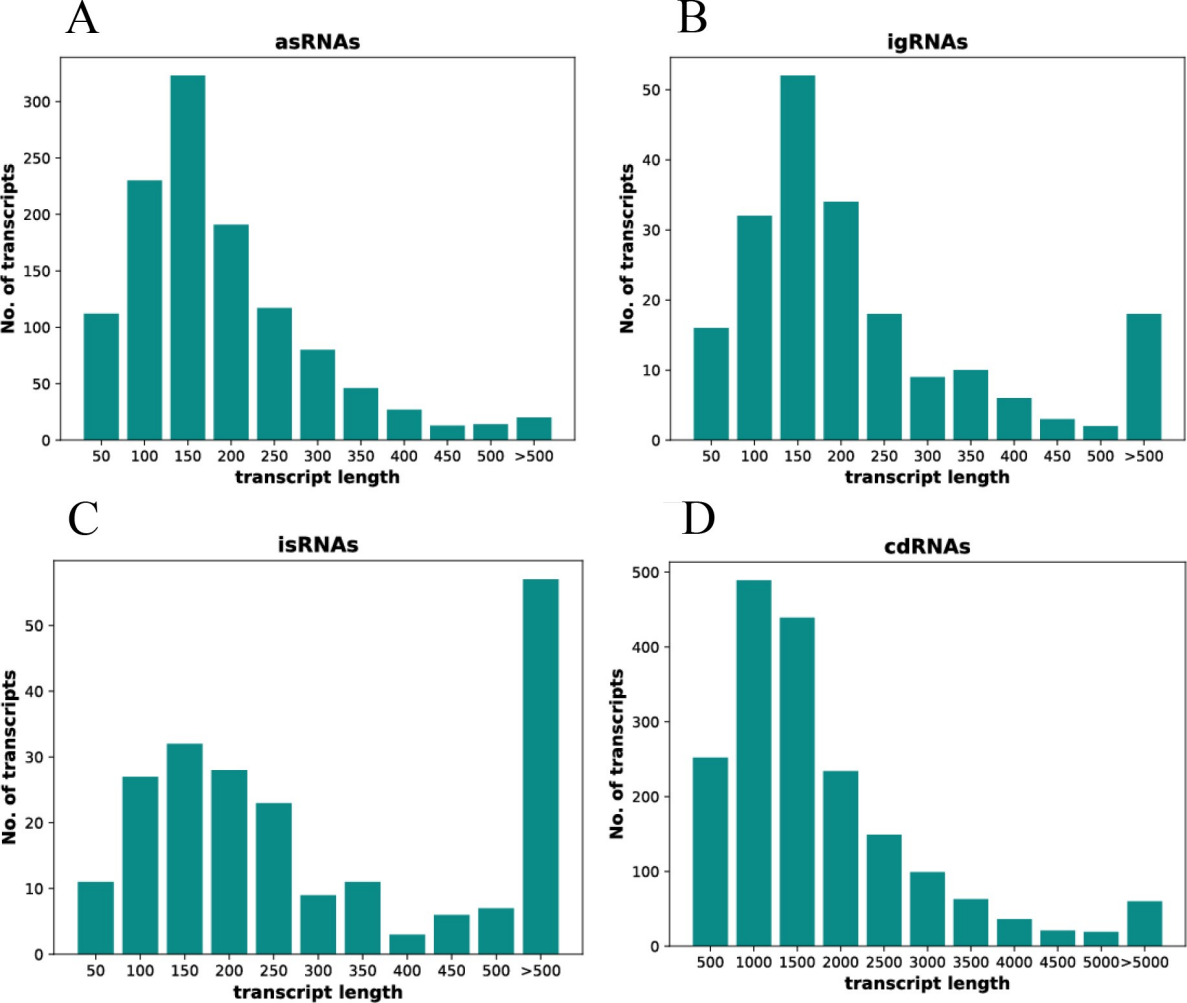
Length distribution of *H*. *volcanii* transcripts. x-axis: RNA length in nt. y-axis: number of RNAs. **A.** asRNAs. **B.** igRNAs. **C.** isRNAs. **D.** cdRNAs. Note that the scaling of the x-axis is very different for the cdRNAs compared to the three classes of sRNAs.

The length distribution of all 1222 asRNAs is shown in Fig 2A. As can be seen, most asRNAs are rather short. More than 70% have lengths between 50 nt and 250 nt, and 86% are shorter than 300 nt. The median size of all asRNAs is 135 nt. Fig 2B shows the length distribution of the 200 igRNAs. Again, most igRNAs are rather short. More than 70% have sizes between 50 nt and 250 nt, and 87% are smaller than 300 nt. The median size is 140 nt. The length distribution of all 213 isRNAs is shown in Fig 2C. Many of the isRNAs are also rather short, and the highest fraction has sizes between 100 nt and 150 nt, like for the other two classes of non-coding RNAs. However, in contrast to the other two classes, the fraction of long isRNAs is considerably higher than for the other two classes. 39% of all isRNAs are longer than 300 nt, and 27% are longer than 500 nt. Therefore, it might be that the isRNAs do not form a coherent group, and that the 61% of isRNAs that are shorter than 300 nt have a different molecular mechanism than the long isRNAs. Taken together, apart from the exception of long isRNAs, the sizes of the three classes of novel non-coding RNAs are rather small and peak between 100 nt and 200 nt. However, all three groups also contain very small RNAs of less than 100 nt. It has been shown previously that *H*. *volcanii* contains very small RNAs, including tRNA-derived fragments (tRFs) [46]. It was revealed that tRFs target the ribosome [47], and a 26 nt fragment of the valin tRNA inhibited translation [48]. Therefore, some very small RNAs might be artifacts of RNA-Seq or might be degradation intermediates, however, it has to be assumed that a significant fraction have important regulatory roles on their own.

The size distribution of all cdRNAs is shown in Fig 2D. The highest fraction of cdRNAs is between 500 nt and 2000 nt. The genome annotation of *H*. *volcanii* includes 575 genes for small proteins of up to 100 amino acids. About 100 cdRNAs were smaller than 300 nt, showing that the genes for a considerable number of these microproteins (μ-proteins) are expressed. However, it also shows that the majority of μ-protein genes were not expressed under any of the four conditions. Also very long cdRNAs were found, 60 cdRNAs were larger than 5000 nt and are indicative of polycistronic operons (the operon structure is discussed below). In summary, the RNA-Seq results gave a conclusive overview of the length distribution of the different classes of non-coding and coding transcripts.

### Comparison of the RNA-Seq with the dRNA-Seq results

Another major goal of the mixed RNA-Seq approach was the detection of additional protein-coding transcripts, which had not been detected in the dRNA-Seq study, where growth was restricted to optimal conditions. In total, the RNA-Seq approach led to the detection of 1855 cdRNAs, about 150 more than the 1702 cdRNAs that were found in the dRNA-Seq study. While 1003 cdRNAs were found in both studies, notably, the results sets of both studies also contained a large number of cdRNAs that were solely found in the respective study. 852 cdRNAs were exclusively found with the mixed RNA-Seq approach (S2 Table). This group should be enriched in transcripts encoding proteins that are not needed under optimal conditions, but under one or more of the additional three conditions. On the other hand, 697 cdRNAs were exclusively detected in the dRNA-Seq study, and this group should be enriched in transcripts with very low levels, which are below the detection limit of the mixed RNA-Seq study.

Comparison of non-coding transcripts is not as straightforward as comparison of cdRNAs, because the dRNA-Seq results do not contain any length information, and the determination of 5’-ends in mixed RNA-Seq has no nucleotide precision. However, it is clear that mixed RNA-Seq also resulted in the identification of novel non-coding RNAs. Mixed RNA-Seq led to the detection of 585 asRNAs to genes, for which no asRNAs had been found by dRNA-Seq. The same is true for 175 isRNAs and 142 igRNAs. Taken together, mixed RNA-Seq identified a high number of cdRNAs as well as non-coding RNAs, which could not be found under optimal conditions, and, thus, which are probably important for one or more of the three non-optimal conditions.

### Elucidation of operon structures

**Fig 3:**
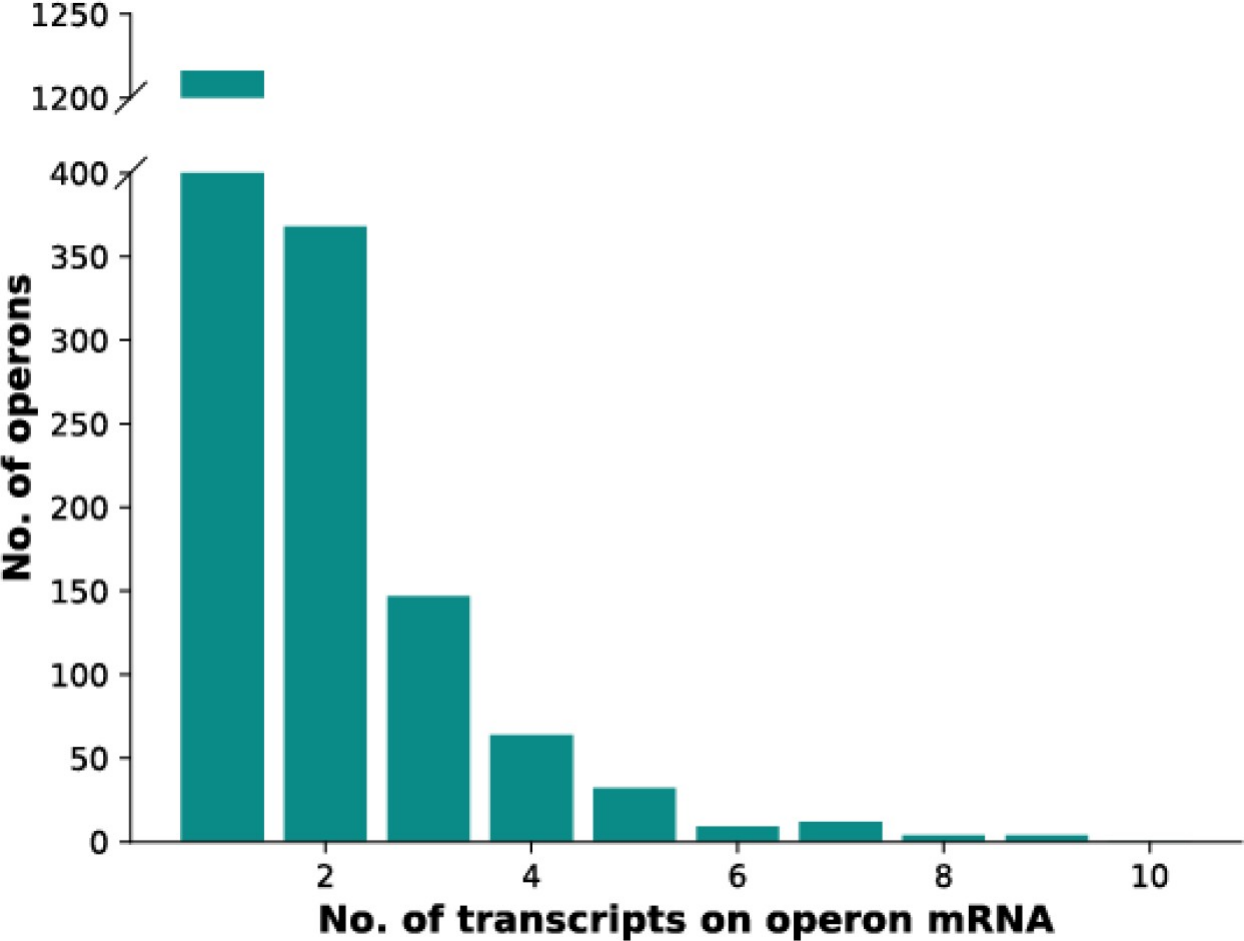
Operon analysis. x-axis: number of ORFs per transcript. y-axis: number of cdRNAs.

Another motivation of the RNA-Seq study was the elucidation of operon structures. The numbers of ORFs predicted to be present on each of the cdRNAs were determined (Fig 3, S2 Table). About 2/3 of the protein-coding transcripts were found to be monocistronic. The second largest fraction was comprised of bicistronic transcripts, and the numbers of transcripts containing three or more ORFs were rather small. 13 transcripts contained more than seven ORFs. These included well-known large operons, e.g. two operons for ribosomal proteins, the *atp* operon encoding the ATP synthase subunits, the *nuo* operon encoding the NADH dehydrogenase-like respiratory complex I, the operon for the phosphotransferase system for sugar import, and an operon encoding RNA polymerase subunits and other genes involved in transcription and translation. One large transcript contained nine genes for the biosynthesis of leucine and isoleucine, and thus the biosynthesis of these amino acids appears to be coupled. Two large transcripts, each with eight ORFs, encoded primarily “conserved hypothetical proteins”, and thus these proteins with unknown functions probably are involved in two common, yet unknown, processes (starting at HVO_0845 and HVO_2323).

Due to the existence of polycistronic transcripts, the number of expressed genes exceeds that of cdRNAs. Table 3 gives an overview of the annotated genes and the ORFs observed on cdRNAs for the four chromosomes. In total, the 1861 cdRNAs encoded 3092 proteins. Thus, a high fraction of 75% of all annotated protein coding genes is expressed under at least one of the four culture conditions. Notably, the fractions of annotated protein-coding genes with observed cdRNAs varied considerably for the four chromosomes (Table 3). While 82% and 74% of all genes from the major chromosome and pHV1 were transcribed under at least one of the four analyzed conditions, this was true for only 56% of all genes from the minor chromosomes pHV3 and pHV4. This indicates that genes are not randomly distributed on the four chromosomes, but that pHV3 and pHV4 are chromosomes that are enriched in genes that are required under specific conditions not represented in the current study. If subsequent studies confirm this finding, *H*. *volcanii* would not only represent a prokaryote with multiple chromosomes [43] and with multiple replication origins on its major chromosome [49], but it would also be an example of a prokaryote with specialized chromosomes. It will be interesting to identify conditions under which the 44% of genes on pHV3 and pHV4 are induced, which are silent under the four conditions tested in this study.

**Table 3:** Fractions of annotated ORFs detected by RNA-Seq.

	Chr	pHV1	pHV3	pHV4	Sum
Annotated ORFs	2992	89	383	636	4100
ORFs on cdRNAs	2453	66	216	357	3092
Fraction (%)	82	74	56	56	75

**Fig 4:**
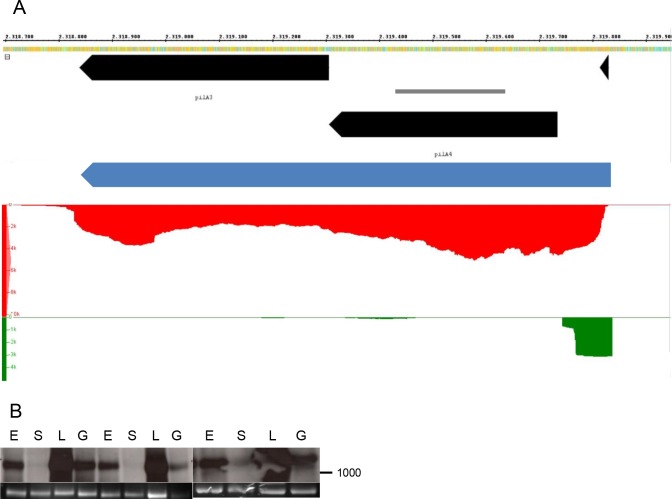
Analysis of a bicistronic operon. **A.** Screenshot from the Integrated Genome Browser. For explanations of panels see Fig 2. **B.** Northern blot analysis. Upper part: hybridization signals. Lower part: 16S rRNA.

Three selected examples of operon transcripts were characterized by Northern blot analyses and showed that the operon analysis can be straightforward and exactly follow the genome annotation, but can also be rather complex. Fig 4 shows the first example, the transcript for the *pilA4-pilA3* operon (HVO_2450 and HVO_2451). The dRNA-Seq study had identified a single TSS upstream of *pilA4*, and the RNA-Seq results showed a rather even distribution of reads over the length of both ORFs. Both analyses were in agreement that the transcript had an extended 5’-UTR of more than 100 nt, which is rare for haloarchaeal transcripts. The Northern blot analysis verified the existence of a binary transcript of the size predicted by RNA-Seq, as well as the absence of monocistronic transcripts for *pilA3* and *pilA4*.

**Fig 5:**
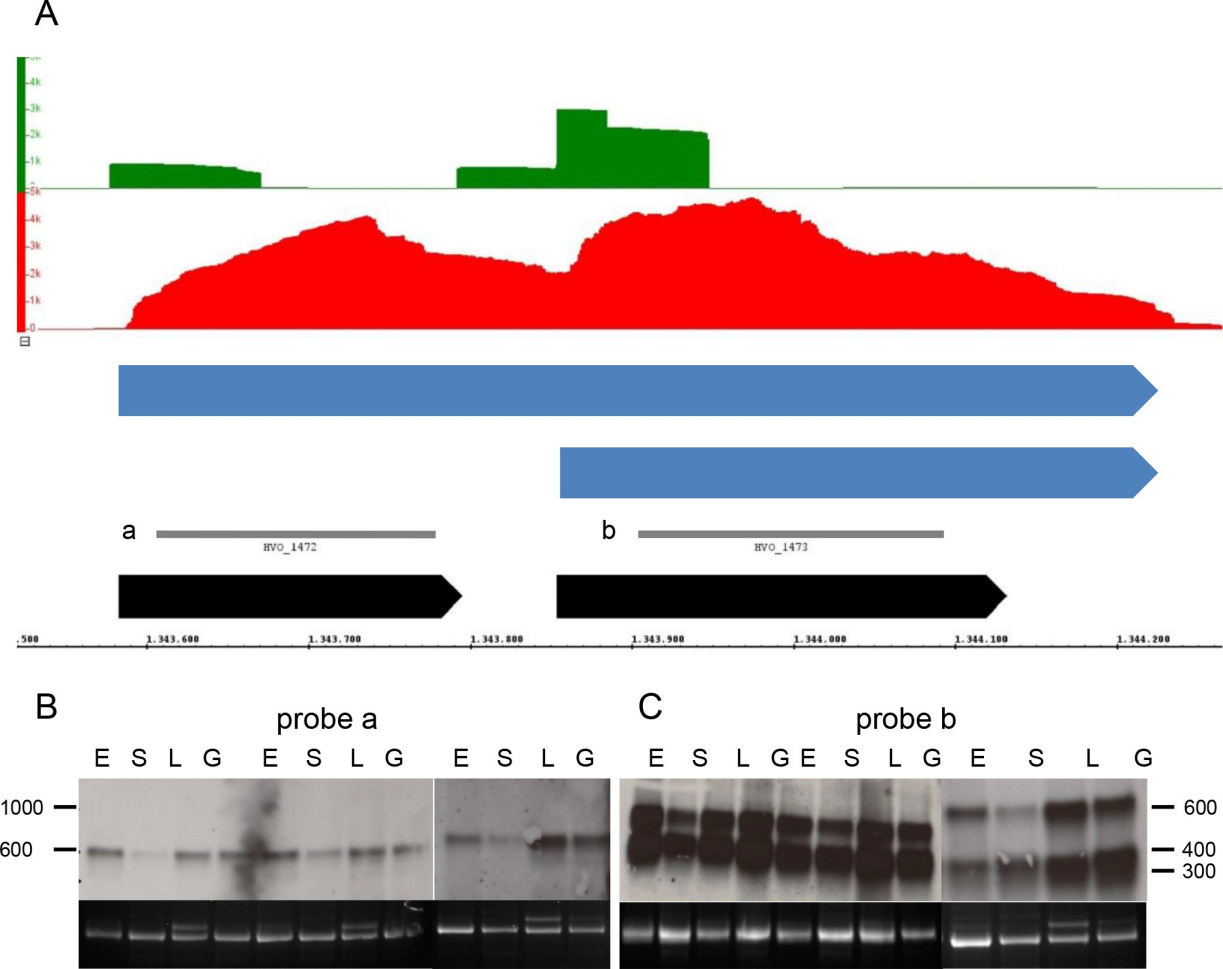
Analysis of two genes with a bicistronic and a monocistronic transcript. **A.** Screenshot from the Integrated Genome Browser. For explanations of panels see Fig 2. In addition, the localization of the two probes a and b is indicated (grey). **B.** Northern blot analysis with probe a. **C.** Northern blot analysis with probe b. A different slightly agarose concentration was used for the third gel in B and C.

The second example is shown in Fig 5. The RNA-Seq results showed reads along the whole length of HVO_1472 and HVO_1473, implying the existence of a bicistronic transcript. However, the dRNA-Seq study had revealed the existence of two TSS, indicating monocistronic transcription of both genes. Northern blot analyses confirmed the correctness of both these seemingly contradictory results. The TSS in front of HVO_1472 led to a formation of a bicistronic transcript (Fig 5B, probe a), while the second TSS resulted in the formation of a monocistronic transcript of HVO_1473. The level of the monocistronic transcript was slightly higher than the level of the bicistronic transcript (Fig 5B, probe b). A similar example has been described recently [50]. The *lsm-rpl37e* operon was found to be transcribed into a bicistronic mRNA from a promoter upstream of the *lsm* gene and into a monocistronic mRNA from a promoter upstream of the *rpl37e* gene. It remains to be analyzed how widespread *H*. *volcanii* makes use of the transcription of genes into monocistronic as well as into polycistronic transcripts. For *H*. *salinarum* a high prevalence of promoters within operons has been described, indicating that formation of more than one transcript from one gene might be typical for haloarchaea [51].

**Fig 6:**
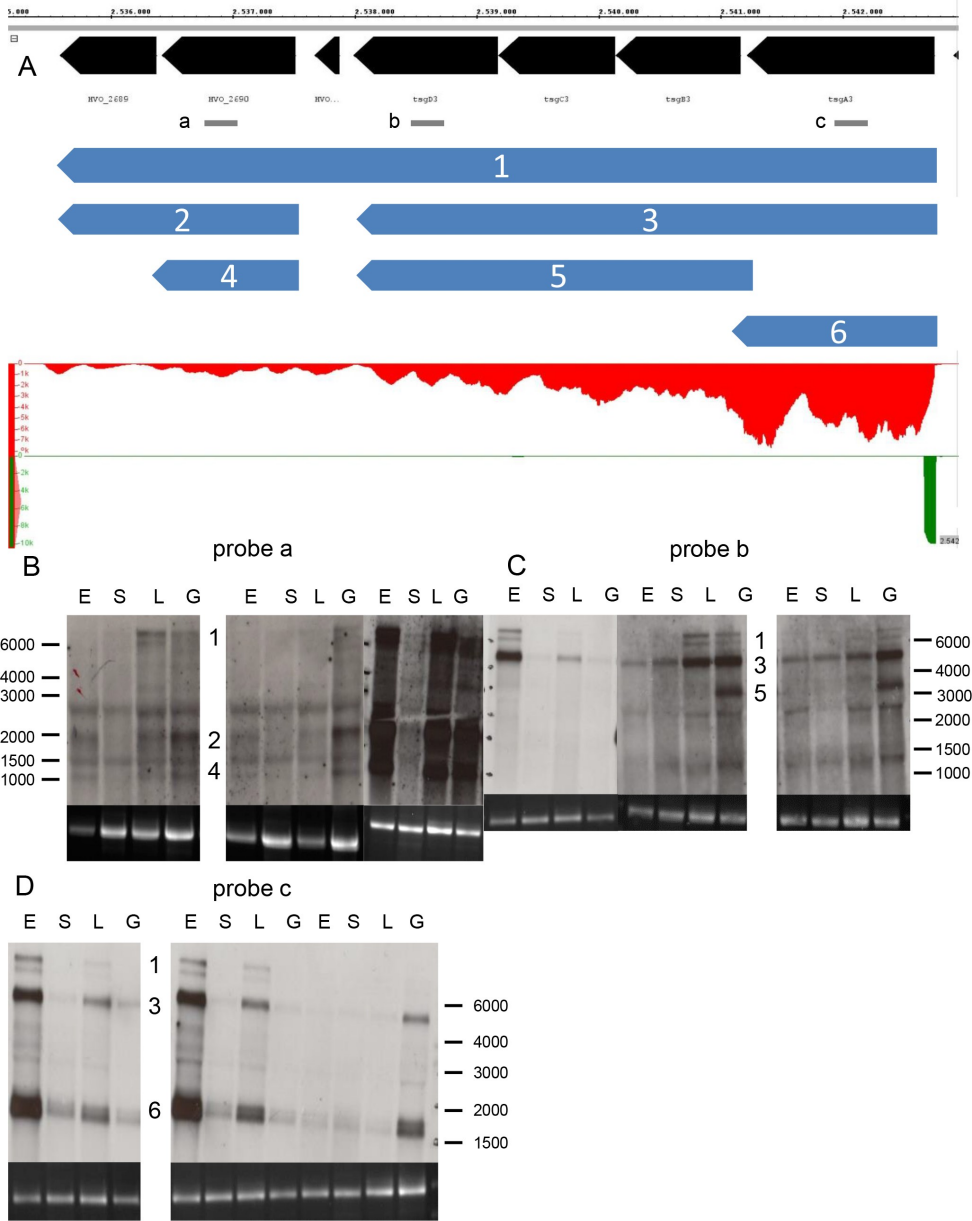
Analysis of a complex gene cluster of seven genes. **A.** Screenshot from the Integrated Genome Browser. For explanations of panels see Fig 2. In addition, the localization of the three probes a, b, and c is indicated (grey). **B.** Northern blot analysis with probe a. **C.** Northern blot analysis with probe b. **D.** Northern blot analysis with probe c. The numbers of the transcripts shown in the overview (A) are shown alongside the respective bands in the Northern blots in B–D.

The third example shows the expression of the *tsg* operon, which is much more complex (Fig 6). The dRNA-Seq study led to the identification of a single TSS in front of *tsgA3*, indicating transcription of the operon into one large polycistronic transcript. However, the RNA-Seq results showed a very uneven distribution of reads along the seven ORFs, with a higher read number at the 5’-ORFs than at the 3’-ORFs. Northern blot analyses revealed the existence of six different transcripts. The existence of a polycistronic transcript containing all seven ORFs was verified with three different probes, however, it had a very low concentration (Fig 6B–6D). A probe specific for *tsgA3* showed that the concentrations of a monicistronic transcript and a tetracistronic transcript (Fig 6D, transcripts 6 and 3) were much higher than that of the longest transcript (Fig 6D, transcript 1). Three additional transcripts were found with two probes specific for *tsgD3* and HVO_2690 (Fig 6B and 6C, probes a and b). Taken together, the results imply that transcription starts at one promoter, and that 1) partial termination and 2) processing of a large polycistronic primary transcript result in the formation of the six transcripts observed by Northern blot analyses. Notably, the levels of the transcripts in Northern blot analyses agree well with the distribution of the read counts of the RNA-Seq analyses. Both show that the monocistronic transcript of the 5’-gene has the highest concentration, followed by the tetracistronic transcript (*tsgA3* –*tsgD3*), followed by the remaining four transcripts. This example shows that the combination of the RNA-Seq and the dRNA-Seq results can indicate the presence of a complex pattern of transcripts, which can then be elucidated by subsequent in depth analyses.

### Very long 5’-UTRs versus upstream sRNAs

The approach of dRNA-Seq has the power to identify TSS. The interpretation is straight-forward when TSS are very close to downstream start codons of annotated genes, then it is clear that the TSS belongs to a cdRNA. However, when the distance between a TSS and a downstream start codon is large, the interpretation becomes ambiguous. The TSS might be indicative for a coding transcript with a very long 5’-UTR, or it might represent a non-coding sRNA localized shortly upstream of the ORF. In dRNA-Seq studies a cutoff has to be used to sort the TSS in either of the two classes, and typically distances of 200 nt or 250 nt are used. The results of RNA-Seq can be used to differentiate between the two ambiguous interpretations of the dRNA-Seq data, and two cases exemplify that both possibilities exist.

S2 Fig shows the results for HVO_2392. The dRNA-Seq study led to the identification of a TSS far upstream of the start codon of the ORF, and it was concluded that the TSS belonged to a intergenic sRNA and it got the gene name HVO_2391s. However, the RNA-Seq results revealed continuous reads from the TSS to a site downstream of the ORF, indicating that the HVO_2392 was transcribed into a cdRNA with a very long 5’-UTR and a 3’UTR of average length. The Northern blot analysis (S2B Fig) verified this interpretation, a single band was found with a size that corresponded to the prediction from the RNA-Seq data.

An opposite example is shown in S3 Fig. Again, the dRNA-Seq data had revealed the existence of a TSS far upstream of an ORF (*orc5*), and it was thus annotated as a sRNA in close proximity of a protein coding gene. In this case the RNA-Seq results underscored this interpretation (S3A Fig, red signals), and the Northern blot analysis verified the existence of a small igRNA and did not give any evidence for an *orc5* mRNA with a long 5’-UTR.

In summary, in *H*. *volcanii* cdRNAs with very long 5’-UTRs as well as igRNAs directly upstream of protein-coding genes exist. The RNA-Seq results can resolve ambiguities that remain if only the annotation and the dRNA-Seq results are taken into account.

### Very long 3’-UTRs, overlapping 3’-UTRs, and differential 3’-UTR formation

The RNA-Seq results also allow an approximate determination of the 3’-UTR length. For a precise determination, a specific technique called “Term-Seq” has to be used that determines the 3’-ends of transcripts with nucleotide resolution. Term-Seq is very new and has as yet only been performed with two archaeal species, i.e. *Sulfolobus solfataricus* and *Methanosarcina mazei* [52].

Nevertheless, the RNA-Seq results already yielded important information about the 3’-length. S4 Fig shows an example of transcript HVO_0416 that, according to RNA-Seq, had an extended 3’-UTR. Northern blot analysis confirmed the existence of a single transcript with a 3’-UTR of more than 100 nt (S4B Fig). Because the ORF is rather small and the transcript does not have a 5’UTR, the 3’-UTR represents about 1/3 of the whole transcript, which implies that the 3’-UTR must have an important function. It may be involved in regulation of RNA stability or regulation of translational efficiency. It has been shown that a considerable fraction of *H*. *volcanii* transcripts shows growth-phase dependent differential translational regulation [53], and that the direction of differential regulation is encoded in the 3’-UTR [54]. It remains to be analyzed whether 3’-UTRs are generally involved in differential translational regulation, like in eukaryotes.

The Term-Seq study of *S*. *solfataricus* and *M*. *mazei* had revealed that more than 30% of all genes have more than one transcriptional terminator and are transcribed into mRNAs with different 3’-UTR lengths. It was hypothesized that the isoforms might be differentially expressed and might be involved in regulation [52]. The formation of RNA isoforms with different 3’-UTRs cannot be analyzed by RNA-Seq. However, the Northern blot analyses uncovered two such examples and indicated that the formation of RNA isoforms might also be typical in *H*. *volcanii*. S5 Fig shows that the gene HVO_2856 is transcribed into two different cdRNAs with very different 3’-UTR lengths (S5A and S5C Fig). Both forms were only produced in stationary phase cells, and the level of the longer form was about one third of the level of the shorter form. S6 Fig shows that isoforms of different lengths not only exist for protein encoding mRNAs, but also for asRNAs (S6A and S6C Fig). In this case differential expression of the two isoforms was observed, i.e. in stationary phase cells the longer form predominated, while, in contrast, in low salt medium the level of the shorter isoform was much higher than that of the longer isoform. It remains to be clarified whether the regulatory functions of the two isoforms indeed differ. Nevertheless, the results show that differential regulation of termination exists in *H*. *volcanii*.

The Term-Seq study had also revealed that 52% of all *S*. *solfataricus* transcripts and 8% of all *M*. *mazei* transcripts have overlapping 3’-ends [52]. Overlapping 3’-ends of cdRNAs also exist in *H*. *volcanii* and S5 Fig shows one example, i.e. the longer version of the transcript HVO_2856 overlaps with the transcript of gene HVO_2855. The RNA-Seq results showed that in total 29% of all cdRNAs had overlapping 3’-UTRs (S2 Table). The high number of overlapping cdRNA pairs considerably extents the number of asRNA/cdRNA pairs and indicates that *H*. *volcanii* makes ample use of RNA-RNA base pair formation for regulatory purposes. The molecular mechanism needs to be clarified in the future, e.g. the identification of the involved double strand-specific or single strand-specific RNases or the importance of regulatory RNA-binding proteins.

### Differential expression under four different conditions

**Fig 7:**
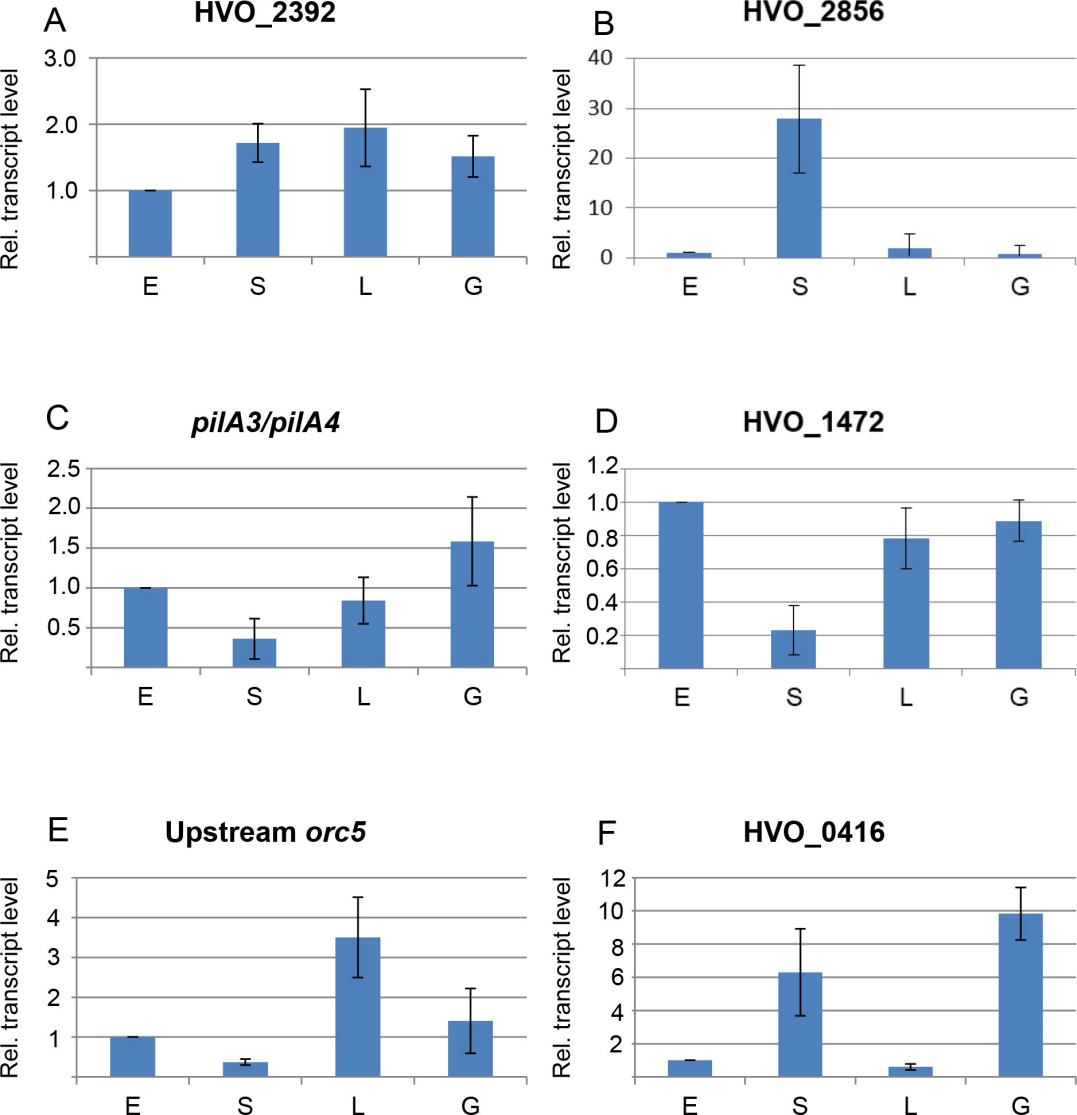
Quantification of transcript levels. The Northern blots were scanned and the signals were quantified with ImageJ. The signals of the four conditions were normalized to the signals of RNAs from exponentially growing cells under optimal conditions (condition E). Average values of three biological replicates and standard deviations are shown. The respective gene designations are shown on the top of each panel.

The transcripts of nearly 20 genes were characterized by Northern blot analyses using RNA from cells cultivated under four different conditions (Fig 1, Figs 4–6, S2–S6 Figs). All transcripts were differentially regulated, indicating that the four conditions were well chosen and the transcriptomes differ considerably in 1) cells growing exponentially under optimal conditions, 2) stationary phase cells, 3) cells growing in low salt, and 4) cells growing in synthetic medium with glucose as carbon source. The transcript levels were quantified using the program ImageJ, and six examples are shown in Fig 7.

Fig 7A shows one of the most constitutive cdRNAs observed with the Northern blot analyses, but even in this case the level differed about twofold in cells growing in the presence of 2.2 M NaCl versus 0.9 M NaCl. In general, the level of the HVO_2392 transcript was higher in slower growing or resting cells than in the fastest growing culture. HVO_2392 encodes a conserved hypothetical protein of unknown function.

Fig 7B shows an example of a transcript that was present (nearly) exclusively in stationary phase cells. HVO_2856 encodes a conserved hypothetical protein, which obviously is not needed during growth, but which is important for resting cells.

In contrast, Fig 7C and 7D show two examples of transcripts with lowest level in stationary phase cells and four- to sixfold higher level in growing cells. This regulatory pattern was surprising for the pilin genes, because haloarchaeal pili are involved in adhesion and biofilm formation [55, 56], and it could have been expected that surface adhesion is more important for stationary phase cells than for exponentially growing planktonic cells. The second example for a gene with lowest transcript level in resting cells was HVO_1472, which encodes a conserved hypothetical protein.

Fig 7E shows another regulatory pattern, namely a transcript that is highly induced solely under low salt condition. It is a sRNA upstream of *orc5*, which encodes one of 16 Origin Recognition Complex (Orc) proteins of *H*. *volcanii*. The reason for the exceeding expansion of the Orc family proteins in *H*. *volcanii* is yet unexplained. Nevertheless, it has been shown that all Orc proteins are involved in copy number regulation of one or more of the four chromosomes [57]. It remains to be clarified whether the sRNA in very close proximity to *orc5* has an influence on *orc5* stability, translational efficiency, or other features, or if the genomic juxtaposition is just by chance.

Fig 7F exemplifies another regulatory pattern, i.e. HVO_0416 encodes a small zinc finger protein with one CPXCG motif, which consists of two appropriately spaced CxxC patterns [40]. A common function for a protein required in resting cells in complex medium and in exponentially growing cells in synthetic medium, but not needed in exponentially growing cells in complex medium, cannot easily be rationalized.

In summary, the results from the Northern blot analyses indicate that differential regulation of transcript levels is typical for *H*. *volcanii*. The comparison of the transcriptomes prior to and after oxidative stress led to the discovery of about 1176 transcripts with differential regulation [18], showing widespread transcript level regulation not only under the four conditions of this study, but also in response to stress. Differential regulation of the transcriptome has also been extensively studied with *H*. *salinarum* [53, 58–62].

## Conclusions

The mixed RNA-Seq results in comparison with previous dRNA-Seq results and with the genome annotation yielded an unprecedented overview of the *H*. *volcanii* transcriptome, e.g. length distributions of all four RNA classes, operon analysis, and approximate 5’-/3’-UTR lengths. Northern blot analyses could exemplify characteristic features of the transcriptome, e.g. complex transcript patterns from gene clusters, differential formation of RNA isoforms, the existence of very long UTRs, and a high fraction of overlapping 3’-ends of cdRNAs. These results are a good starting point for further analyses, aiming to more deeply unravel RNA-based regulation in haloachaea.

## Methods

### Strain, media, and growth conditions

*Haloferax volcanii* strain H26 (Δ*pyrE2*) was obtained from Thorsten Allers (Nottingham). It was grown in complex medium [63] with either 2.2 M NaCl (optimal conditions) or with 0.9 M NaCl (low salt condition), as indicated in the text. The salt concentration of 0.9 M is very close to 0.7 M, which is the lowest limit that allows growth of *H*. *volcanii* [64]. It should be noted that only the NaCl concentration was lowered while all other ion concentrations remained unchanged. H26 was also grown in synthetic medium with 0.5% (w/v) glucose as sole carbon and energy source and with 10 mM NH_4_Cl as nitrogen source [65]. 30 ml cultures were grown in 100 ml Erlenmeyer flasks at 42°C with good aeration (250 rpm). Culture growth was monitored spectroscopically at 600 nm. The cultures were inoculated with exponentially growing pre-cultures, which had been cultivated under identical conditions. They were grown to mid-exponential growth phase (OD_600_ 0.5) or stationary phase (OD_600_ 1.2), before they were harvested by centrifugation and used for RNA isolation.

### RNA Isolation and mixed RNA-Seq

Cultures were grown under four different conditions, as described in the text and summarized in Table 1. From each culture, 5 x 10^8^ cells were harvested by centrifugation. Total RNA was isolated using the NucleoSpin miRNA Kit of Macherey-Nagel (Düren, Germany) according to the manufacturer’s instructions. The isolation procedure included an on-column DNase digestion. The integrity of the RNA was verified by analytical agarose gel electrophoresis. A PCR analysis revealed that the samples contained small amounts of DNA. Therefore, a second purification procedure with an on-column DNase digestion step was performed. After that, an extensive PCR analysis (40 cycles) revealed that the samples were totally free of DNA. For each of the four preparations, 1.5 μg was used to generate a pool of 6 μg of mixed RNA. The mixed RNA was sent to StarSeq (Mainz, Germany), where RNA-Seq was performed. First, rRNA was depleted, then 50 million paired end reads with read lengths of 150 nt were generated using an Illumina NextSeq500^TM^ system. It should be noted that the sequencing represents only one technical replicate, however, 50 million reads adequately represent the transcripts of a genome of 4 Mbp. The number of biological replicates cannot be clearly defined for this mixed RNA-Seq approach, because each transcript can be present in one to four of the cultures grown under four different conditions. The mixed sample does not contain any information about (differential) transcript levels under the four different conditions. However, as discussed above, comparison with the previous dRNA-Seq results can identify transcripts that maybe present only under non-optimal conditions (detected by mixed RNA-Seq, but not by RNA-Seq) and transcripts that might have a low level (detected by dRNA-Seq, but not by mixed RNA-Seq).

### Data analysis

Removal of adapter sequences and of low quality nucleotides was performed with cutadapt version 1.9.1 [66]. The reverse complement of the reads were generated using the command 2fastx_reverse_complement” of the fastx toolkit version 0.0.13 (http://hannonlab.cshl.edu/fastx_toolkit/). Reads were mapped using READemption’s (version 0.4.2, [67] subcommand “align” and segemehl 0.2.0 [68] requiring a mapping accuracy of 100%. This lead the successful mapping of 40,712,641 of the total 58,437,942 reads (69.67%). The alignments where translated into strand specific coverage files in wiggle format using READemption’s subcommand “coverage”. Transcript were predicted using ANNOgesic [27] subcommand “transcript” requiring a minimal coverage of 70 reads while allowing gaps of lower coverage that were spanned by ORFs. The cut-off value of 70 reads was chosen after the careful inspection of the predictions result with value ranging from 5–105 reads. Overlapping paired-end reads for annotated transcripts where calculated with htseq-count (part of the 'HTSeq' framework, version 0.9.1) [69] after extracting the pairs from the mapping file in BAM format using samtools view [70]. All READemption and ANNOgesic input and output files including a Unix Shell script to run the analysis are deposited at Zenodo at https://doi.org/10.5281/zenodo.1434894.

### Northern blot analysis

RNA isolation, Northern blot analysis, and probe generation were essentially performed as described previously [71]. In short, for each sample 4 μg of total RNA were separated on denaturing formaldehyde gels. Concentrations of 1% (w/v) or 2% (w/v) agarose were used, depending on the expected transcript length. The RNAs were transferred to Nylon membranes by capillary blotting and were fixed by UV-crosslinking. Digoxigenin labeled DNA probes were generated by PCR using Dig-dUTP and a dNTP mix with reduced dTTP concentration. The primers used for probe generation are summarized in S4 Table. Hybridization was performed overnight at 50°C. The membranes were washed twice in 2xSCC/0.1% (w/v) SDS and twice in 1xSCC/0.1% (w/v) SDS. DIG detection was performed with an alkaline phosphatase coupled anti-DIG antibody and the chemiluminescence substrate CDP-star according to the manufacturer`s instruction (Roche, Mannheim, Germany). The signals were visualized on X-ray films (GE Healthcare, Buckinghamshire, UK). The films were scanned and the signals were quantified using the program ImageJ (http://rsbweb.nih.gov/ij/). Three biological replicates were performed, and averages and standard deviations were calculated. The full uncropped Northern blots are shown in S7 Fig, the informative parts of the Northern blots are shown in the Figs discussed in the text.

### Programs and databases

Bioinformatic analyses of the *H*. *volcanii* genome were performed at the website Halolex [72]. The Halolex database is freely available, but currently usage is restricted to registered users. To request access, send a mail to halolex@rzg.mpg.de. The annotation of the *H*. *volcanii* genome gets constantly updated. Every few years the updated annotation is transferred to Genbank and Uniprot (last 2016), so that the updated annotation is available in common public databases.

The Integrated Genome Browser [73] was used to visualize the genome annotation as well as the results of the dRNA-Seq study and this mixed RNA-Seq study. The program Codonpreference was used to identify regions that exhibit the *H*. *volcanii* codon usage and that are thus probably protein-coding [45]. The functional categories of proteins were taken from the Halolex database.

## Supporting information

S1 Table Results of previous RNA-Seq and dRNA-Seq studies with archaea. (DOC)

S2 Table Summary of results of the RNA-Seq study. The Table lists the predicted RNAs, their genomic locations and their lengths. It also includes the associated HVO_number (gene identifier), presence of overlapping 3’-UTRs, and number of genes on polycistronic transcripts. For cdRNAs, the identification in the previous dRNA-Seq study is flagged [19].
(XLSX)

S3 Table Comparison of three codon usage tables: 1) the codon usage table computed from the more than 4000 protein coding genes of *H*. *volcanii*, 2) the codon usage table computed from the 1077 small proteins of up to 150 amino acids from *H*. *volcanii*, and 3) the codon usage table for *E*. *coli*. (DOC)

S4 Table Oligonucleotides that were used to generate probes for the Northern blot analyses. (DOC)

S1 Fig Examples of RNA-Seq results and the comparison with the genome annotation and dRNA-Seq results. Screenshots from the Integrated Genome Browser are shown. The upper half shows results from the top strand, the lower half from the bottom strand. The middle line represents the genome sequence, genome positions are indicated. The following data are shown in the panels from the genome line to the top/bottom: gene annotations (blue), dRNA-Seq results (green), RNA-Seq results (red), and transcript prediction based on the RNA-Seq results (blue).
**A.** Example of a gene with continuous reads over the whole length of the transcript. **B.** Example of a gene with dis-continuous reads over the length of the transcript. **C.** Example of a region with four transcribed protein-coding genes and one asRNA. **D.** Example of a region with non-transcribed genes.
(PPTX)

S2 Fig Detection of a very long 5’-UTR (rather than an upstream sRNA). **A.** Screenshot from the Integrated Genome Browser. For explanations of panels see Fig 2. **B.** Northern blot analysis.
(PPTX)

S3 Fig Detection of an upstream sRNA (rather than a very long 5’-UTR). **A.** Screenshot from the Integrated Genome Browser. For explanations of panels see Fig 2. **B.** Northern blot analysis.
(PPTX)

S4 Fig Detection of a very long 3’-UTR. A. Screenshot from the Integrated Genome Browser. For explanations of panels see Fig 2. **B.** Northern blot analysis.
(PPTX)

S5 Fig Example for one gene that is transcribed into two transcript isoforms of different lengths and example for overlapping 3’-ends of RNAs. **A.** Screenshot from the Integrated Genome Browser. For explanations of panels see Fig 2. **B.** Northern blot analysis with probe a. **C.** Northern blot analysis with probe b.
(PPTX)

S6 Fig Example for two isoforms of an asRNA. **A.** Screenshot from the Integrated Genome Browser. For explanations of panels see Fig 1. **B.** Northern blot analysis with probe a. **C.** Northern blot analysis with probe b.
(PPTX)

S7 Fig Complete uncropped versions of all Northern blots shown in the Figs 1 and 5–7, and S2–S6. (PPTX)

## Abbreviations

dRNA-Seq: differential RNA-Seq
ORF: open reading frame
UTR: untranslated region.
