# Supplementary material for: Characterization of the transcriptome of Haloferax volcanii, grown under four different conditions, with mixed RNA-Seq
Source: PLoS One. 2019 Apr 30;14(4):e0215986. doi: 10.1371/journal.pone.0215986 (PMC6490895; doi:10.1371/journal.pone.0215986)
Supplement: S1 Table — (DOC) [file pone.0215986.s001.doc]

Supplementary Table S1. Results of previous (d)RNA-Seq studies with archaeal species. The transcripts were divided into the classes protein-coding transcripts (cdRNAs), cis antisense transcripts (asRNAs), internal sense transcripts (isRNAs), and intergenic transcripts (igRNAs). Some data were not recorded (n.r.)

| Species | No. of  ORFs | cdRNAs | asRNAs | isRNAs | igRNAs | Type | Ref. |
| --- | --- | --- | --- | --- | --- | --- | --- |
| *Haloferax volcanii* | 4040 | 1851 | 1244 | 1153 | 395 | dRNA-Seq | [14] |
| *Methanolobus*  *psychrophilus* | 3167 | 2735 | 1110 | 1440 | 195 | dRNA-Seq | [16] |
| *Methanosarcina mazei* | 3371 | 586 | 43 | n.r. | 199 | dRNA-Seq | [20] |
| *Pyrococcus abyssi* | 1784 | 1893 | 215 | n.r. | 107 | RNA-S. | [18] |
| *Sulfolobus solfataricus* | 2994 | >1000 | 185 | n.r. | 125 | dRNA-Seq | [19] |
| *Thermococcus kodakaensis* | 2306 | 1254 | 1018 | 644 | 69 | dRNA-Seq | [17] |
