# Supplementary material for: Characterization of the transcriptome of Haloferax volcanii, grown under four different conditions, with mixed RNA-Seq
Source: PLoS One. 2019 Apr 30;14(4):e0215986. doi: 10.1371/journal.pone.0215986 (PMC6490895; doi:10.1371/journal.pone.0215986)
Supplement: S3 Table — (DOC) [file pone.0215986.s003.doc]

S3 Table. Codon usage tables for *H. volcanii*, computed from the 4074 protein-coding genes annotated in the genome of *H. volcanii* and from the 1077 small proteins of up to 150 amino acids. For comparison, the codon usage table of *E. coli* is also included. Large differences between the two species are indicated in red (*H. volcanii*) and blue (*E. coli*).

| Amino acid | Codon | Fraction  *H. volcanii*  all proteins | Fraction  *H. volcanii*  small proteins  <150 aa | Fraction  *E. coli*  all proteins |
| --- | --- | --- | --- | --- |
| Gly | GGG | 0.19 | 0.19 | 0.15 |
| Gly | GGA | 0.09 | 0.12 | 0.11 |
| Gly | GGU | 0.11 | 0.14 | 0.34 |
| Gly | GGC | 0.61 | 0.55 | 0.40 |
| Glu | GAG | 0.68 | 0.63 | 0.31 |
| Glu | GAA | 0.32 | 0.37 | 0.69 |
| Asp | GAU | 0.08 | 0.10 | 0.63 |
| Asp | GAC | 0.92 | 0.90 | 0.37 |
| Val | GUG | 0.23 | 0.24 | 0.37 |
| Val | GUA | 0.02 | 0.03 | 0.15 |
| Val | GUU | 0.06 | 0.08 | 0.26 |
| Val | GUC | 0.69 | 0.65 | 0.22 |
| Ala | GCG | 0.47 | 0.46 | 0.36 |
| Ala | GCA | 0.06 | 0.08 | 0.21 |
| Ala | GCU | 0.04 | 0.06 | 0.16 |
| Ala | GCC | 0.43 | 0.40 | 0.27 |
| Arg | AGG | 0.02 | 0.02 | 0.02 |
| Arg | AGA | 0.03 | 0.04 | 0.04 |
| Ser | AGU | 0.06 | 0.07 | 0.15 |
| Ser | AGC | 0.22 | 0.23 | 0.28 |
| Lys | AAG | 0.71 | 0.67 | 0.23 |
| Lys | AAA | 0.29 | 0.33 | 0.77 |
| Asn | AAU | 0.09 | 0.10 | 0.45 |
| Asn | AAC | 0.91 | 0.90 | 0.55 |
| Met | AUG | 1.00 | 1.00 | 1.00 |
| Ile | AUA | 0.06 | 0.07 | 0.07 |
| Ile | AUU | 0.16 | 0.19 | 0.51 |
| Ile | AUC | 0.78 | 0.75 | 0.42 |
| Thr | ACG | 0.47 | 0.46 | 0.27 |
| Thr | ACA | 0.05 | 0.07 | 0.13 |
| Thr | ACU | 0.05 | 0.06 | 0.17 |
| Thr | ACC | 0.44 | 0.41 | 0.44 |
| Trp | UGG | 1.00 | 1.00 | 1.00 |
| End | UGA | 0.00 | 0.00 | 0.29 |
| Cys | UGU | 0.39 | 0.39 | 0.45 |
| Cys | UGC | 0.61 | 0.61 | 0.55 |

| End | UAG | 0.00 | 0.00 | 0.07 |
| --- | --- | --- | --- | --- |
| End | UAA | 0.00 | 0.00 | 0.64 |
| Tyr | UAU | 0.10 | 0.13 | 0.57 |
| Tyr | UAC | 0.90 | 0.87 | 0.43 |
| Leu | UUG | 0.05 | 0.06 | 0.13 |
| Leu | UUA | 0.01 | 0.02 | 0.13 |
| Phe | UUU | 0.09 | 0.11 | 0.57 |
| Phe | UUC | 0.91 | 0.89 | 0.43 |
| Ser | UCG | 0.43 | 0.39 | 0.15 |
| Ser | UCA | 0.04 | 0.05 | 0.12 |
| Ser | UCU | 0.04 | 0.05 | 0.15 |
| Ser | UCC | 0.21 | 0.20 | 0.15 |
| Arg | CGG | 0.26 | 0.26 | 0.10 |
| Arg | CGA | 0.13 | 0.17 | 0.06 |
| Arg | CGU | 0.06 | 0.07 | 0.38 |
| Arg | CGC | 0.50 | 0.44 | 0.40 |
| Gln | CAG | 0.80 | 0.77 | 0.65 |
| Gln | CAA | 0.20 | 0.23 | 0.35 |
| His | CAU | 0.08 | 0.10 | 0.57 |
| His | CAC | 0.92 | 0.90 | 0.43 |
| Leu | CUG | 0.23 | 0.24 | 0.50 |
| Leu | CUA | 0.02 | 0.02 | 0.04 |
| Leu | CUU | 0.05 | 0.05 | 0.10 |
| Leu | CUC | 0.65 | 0.62 | 0.10 |
| Pro | CCG | 0.53 | 0.53 | 0.52 |
| Pro | CCA | 0.05 | 0.07 | 0.19 |
| Pro | CCU | 0.03 | 0.04 | 0.16 |
| Pro | CCC | 0.39 | 0.36 | 0.12 |
