# Supplementary material for: Characterization of the transcriptome of Haloferax volcanii, grown under four different conditions, with mixed RNA-Seq
Source: PLoS One. 2019 Apr 30;14(4):e0215986. doi: 10.1371/journal.pone.0215986 (PMC6490895; doi:10.1371/journal.pone.0215986)
Supplement: S4 Table — (DOC) [file pone.0215986.s004.doc]

Supplementary Table S3. List of primers that were used to generate the probes for the Northern blot analyses.

| **Locus Taq** | **Gene Name** | **5´ Primer** | **3´Primer** |
| --- | --- | --- | --- |
| **HVO_2451** | *pilA4* | AAGACCGTGCAGTCAGTCC | GTTTACAACGGCGGACCCAC |
| **HVO_2392** | *chp* | CGGCCGCACCTCACAGTTC | TGCCGCGGTCAACCGTAATC |
| **us HVO_1725** | Upstream *orc5* | TTCGGAGTTTGCGAGTCAG | CACACGAAACGGAGGTATTG |
| **HVO_1725** | *orc5* (asRNA) | CGGGAGCGCGGAGGGAAGC | TGCGGTGGTACGGCGTTCCC |
| **HVO_1725** | *orc5* (asRNA) | CGAACGAGCCGCGTCTAC | CCGCTGTGTTTCGACTGCTC |
| **HVO_2690** | gfo family reductase | AGCGGCCCTCCTCGTAGTGG | CGTCGGCGGCCTCGACATTC |
| **HVO_2692** | *tsgD3* | CACGTCTTCCGGGCGGATGC | CCGAACTCCAGCGCCTGCAC |
| **HVO_2695** | *tsgA3* | AGGAGGGTCGGTTCTTCAC | GACAACGGTGACAACGGTAG |
| **HVO_0850** | *panA* (asRNA) | CGGCCTTGGCGAGCATCGTC | ATGCAGGAGGTCCGCGAGACG |
| **HVO_1472** | *chp* | GCCCAAAGTAGAGATTACCG | GACGTACTCGTCTTCGTG |
| **HVO_1473** | UPF0058 family protein | ACTGCTGGAGCTGCACGAAC | GGCGTCGTCGGCCAACTC |
| **HVO_2855** | *chp* | CGACGATGGTACGCATCGC | TAGAACCCGGACGGAAGACG |
| **HVO_2856** | *chp* | AGTCGCTCGCGGACTCGTTC | ACGGCTGCACCAACTGACTC |

Chp: conserved hypothetical protein; us: upstream; UPF: uncharacterized protein family
