# Supplementary figures and images for: Characterization of the transcriptome of Haloferax volcanii, grown under four different conditions, with mixed RNA-Seq
Source: PLoS One. 2019 Apr 30;14(4):e0215986. doi: 10.1371/journal.pone.0215986 (PMC6490895; doi:10.1371/journal.pone.0215986)

## Slide 1
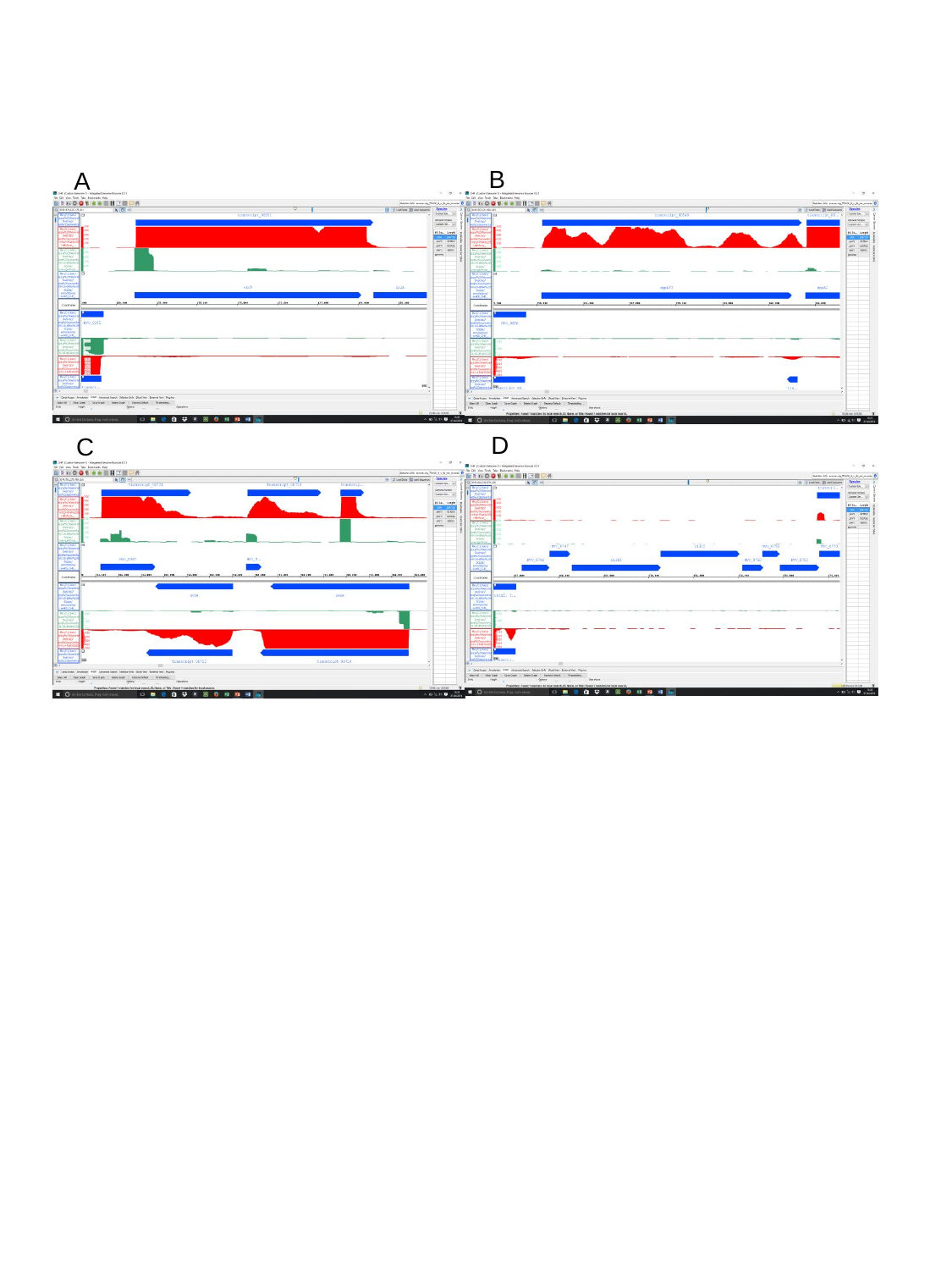

B
A
D
C

Supplement: S1 Fig — Screenshots from the Integrated Genome Browser are shown. The upper half shows results from the top strand, the lower half from the bottom strand. The middle line represents the genome sequence, genome positions are indicated. The following data are shown in the panels from the genome line to the top/bottom: gene annotations (blue), dRNA-Seq results (green), RNA-Seq results (red), and transcript prediction based on the RNA-Seq results (blue). A. Example of a gene with continuous reads over the whole length of the transcript. B. Example of a gene with dis-continuous reads over the length of the transcript. C. Example of a region with four transcribed protein-coding genes and one asRNA. D. Example of a region with non-transcribed genes. (PPTX) [file pone.0215986.s005.pptx]

## Slide 1
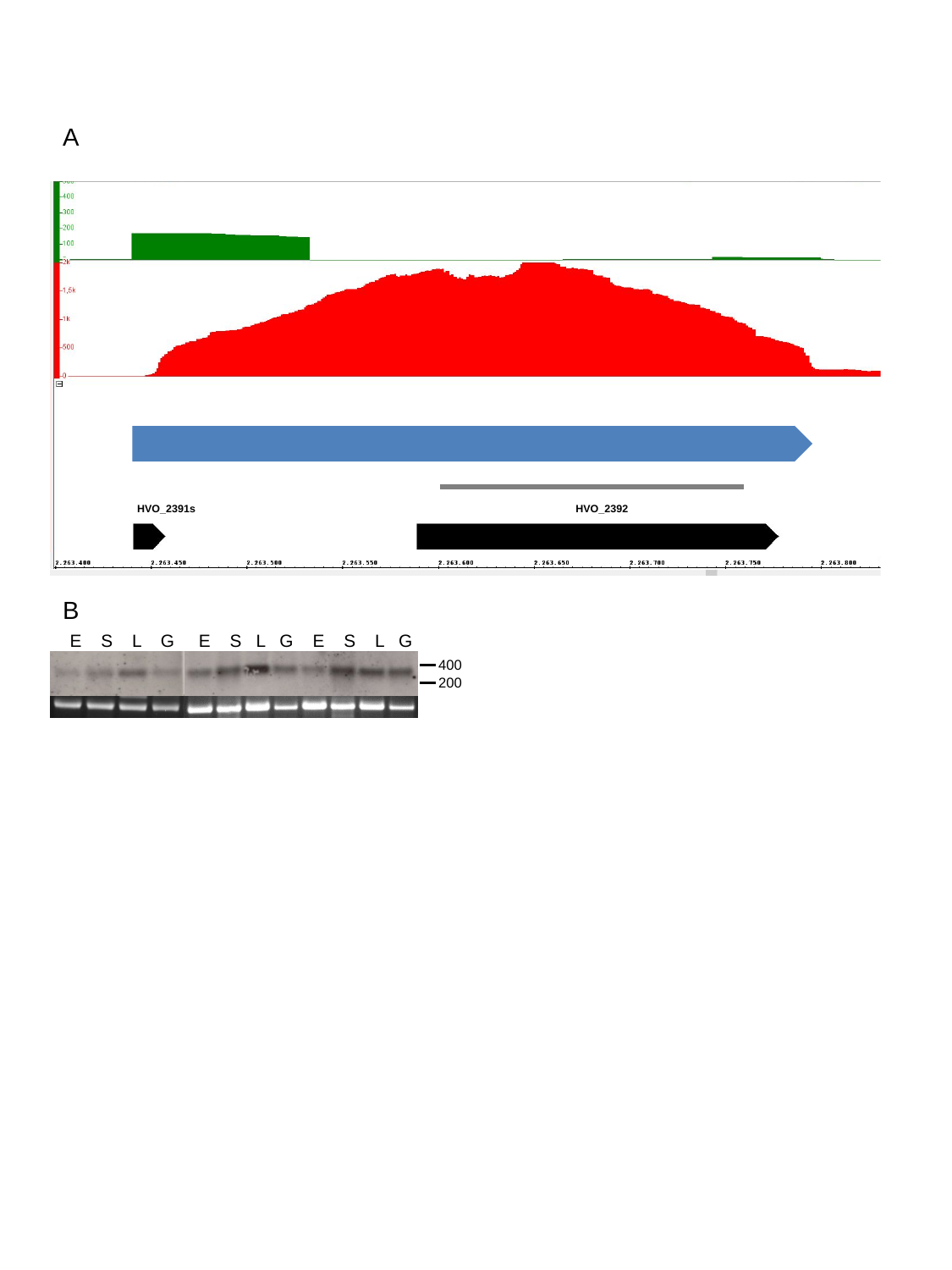

A
HVO_2391s
HVO_2392
B
E S L G E S L G E S L G
400
200

Supplement: S2 Fig — A. Screenshot from the Integrated Genome Browser. For explanations of panels see Fig 2. B. Northern blot analysis. (PPTX) [file pone.0215986.s006.pptx]

## Slide 1
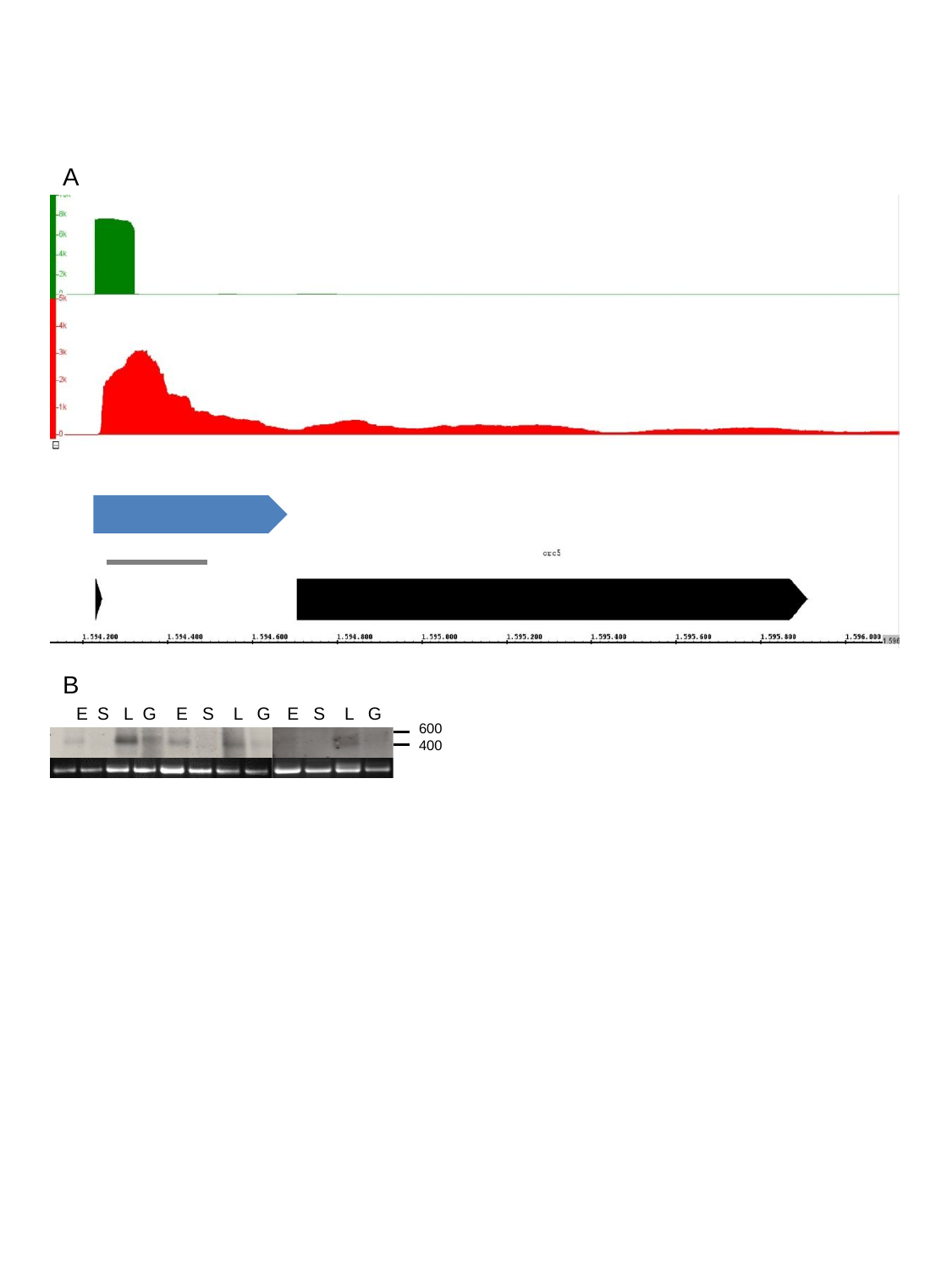

A
B
E S L G
E S L G
E S L G
600
400

Supplement: S3 Fig — A. Screenshot from the Integrated Genome Browser. For explanations of panels see Fig 2. B. Northern blot analysis. (PPTX) [file pone.0215986.s007.pptx]

## Slide 1
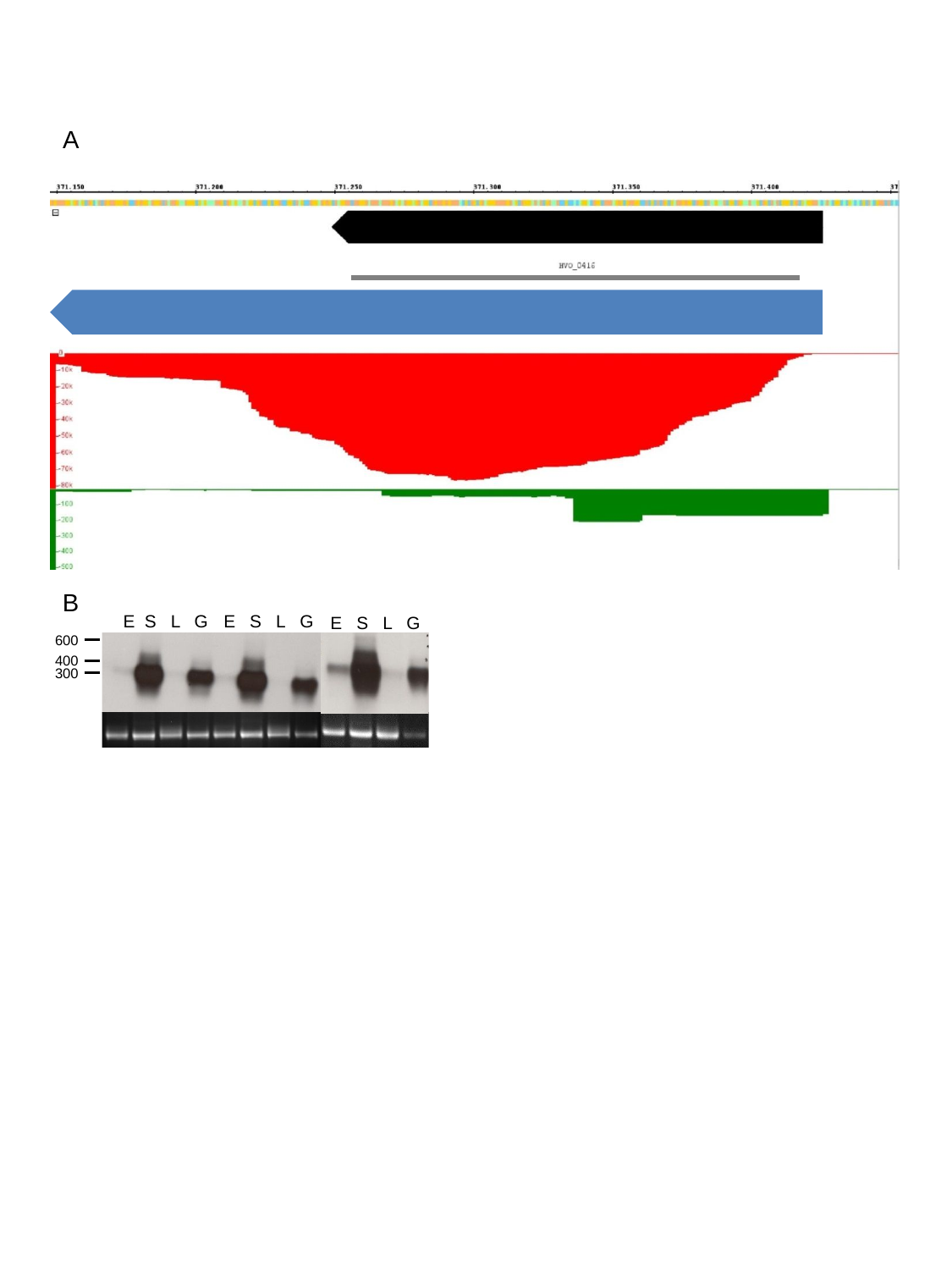

A
B
E S L G
E S L G
E S L G
600
400
300

Supplement: S4 Fig — For explanations of panels see Fig 2. B. Northern blot analysis. (PPTX) [file pone.0215986.s008.pptx]

## Slide 1
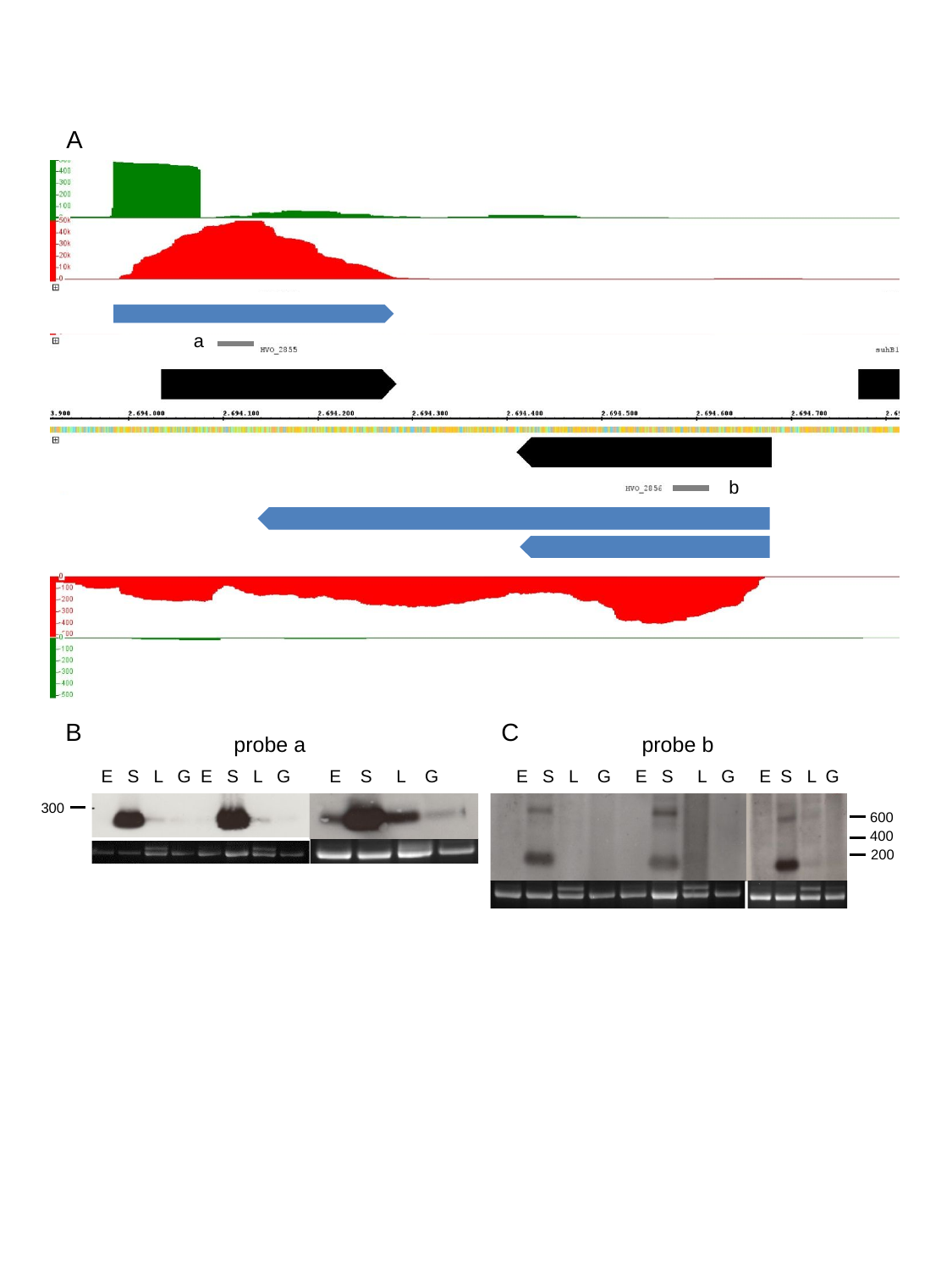

A
a
b
B
C
probe a
probe b
E S L G E S L G E S L G
E S L G E S L G E S L G
300
600
400
200

Supplement: S5 Fig — A. Screenshot from the Integrated Genome Browser. For explanations of panels see Fig 2. B. Northern blot analysis with probe a. C. Northern blot analysis with probe b. (PPTX) [file pone.0215986.s009.pptx]

## Slide 1
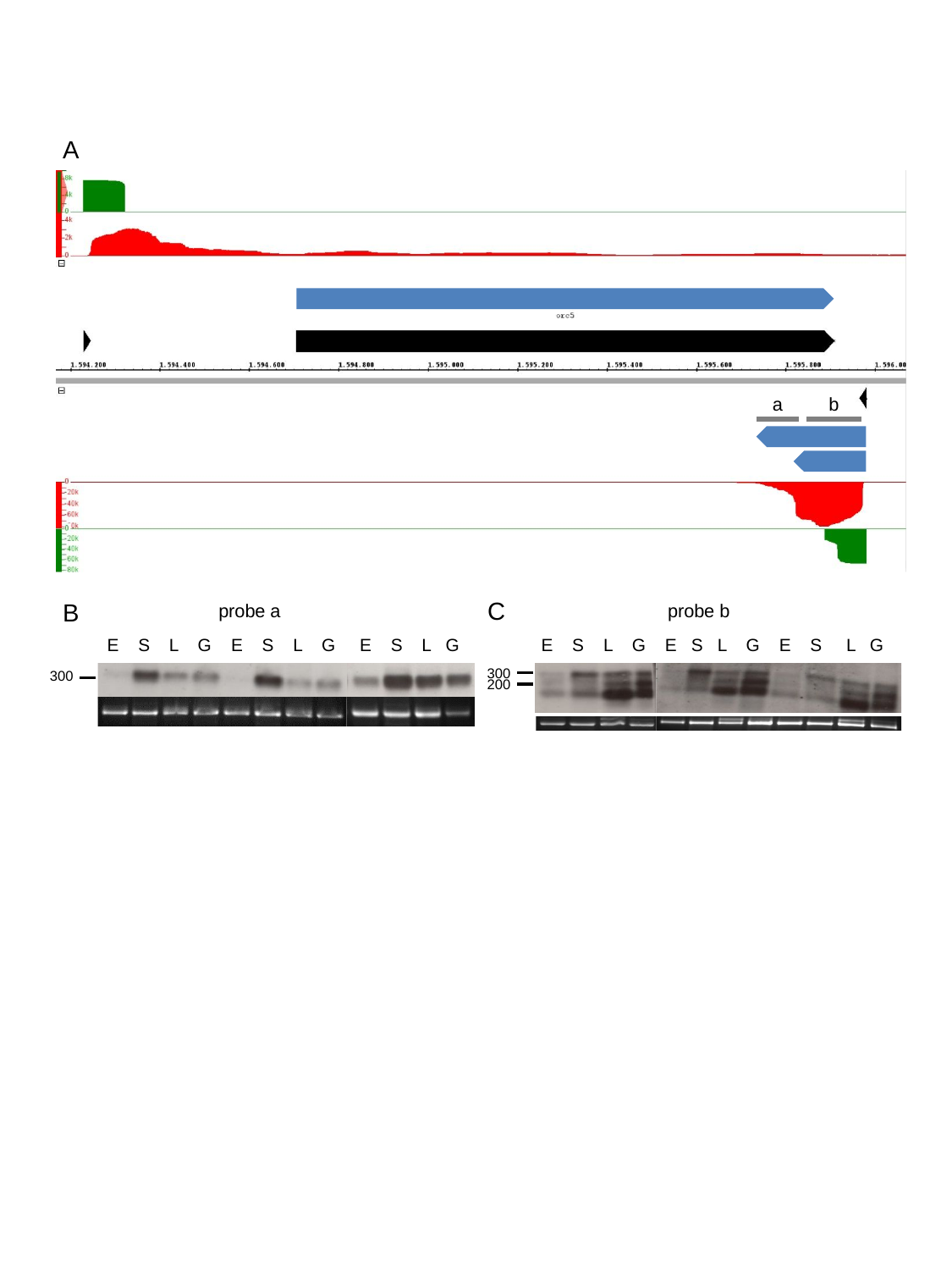

A
a
b
C
B
probe a
probe b
E S L G E S L G E S L G
E S L G E S L G E S L G
300
300
200

Supplement: S6 Fig — A. Screenshot from the Integrated Genome Browser. For explanations of panels see Fig 1. B. Northern blot analysis with probe a. C. Northern blot analysis with probe b. (PPTX) [file pone.0215986.s010.pptx]
