# Supplementary material for: Characterization of the transcriptome of Haloferax volcanii, grown under four different conditions, with mixed RNA-Seq
Source: PLoS One. 2019 Apr 30;14(4):e0215986. doi: 10.1371/journal.pone.0215986 (PMC6490895; doi:10.1371/journal.pone.0215986)
Supplement: S7 Fig — (PPTX) [file pone.0215986.s011.pptx]

## Slide 1
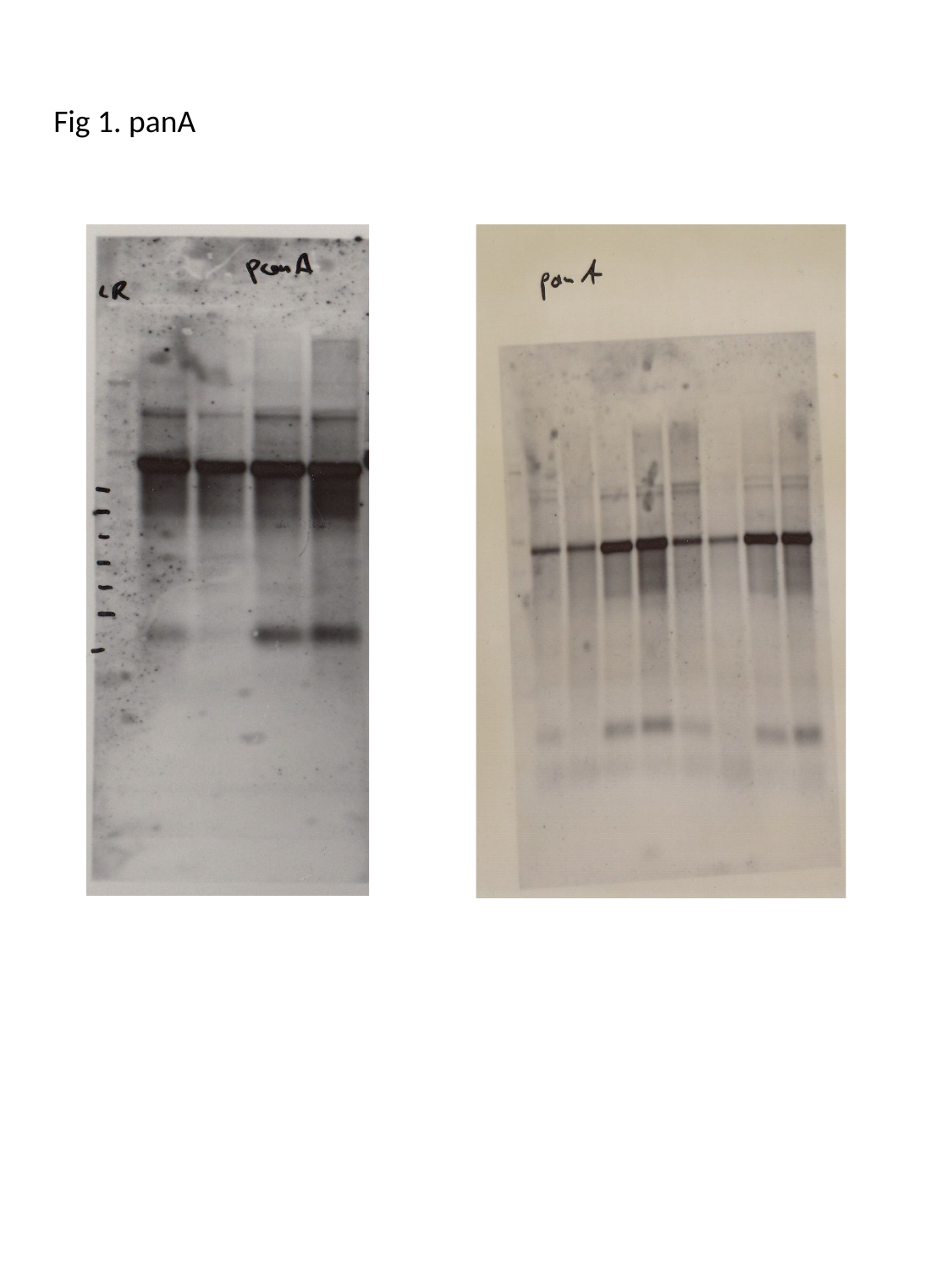

Fig 1. panA

## Slide 2
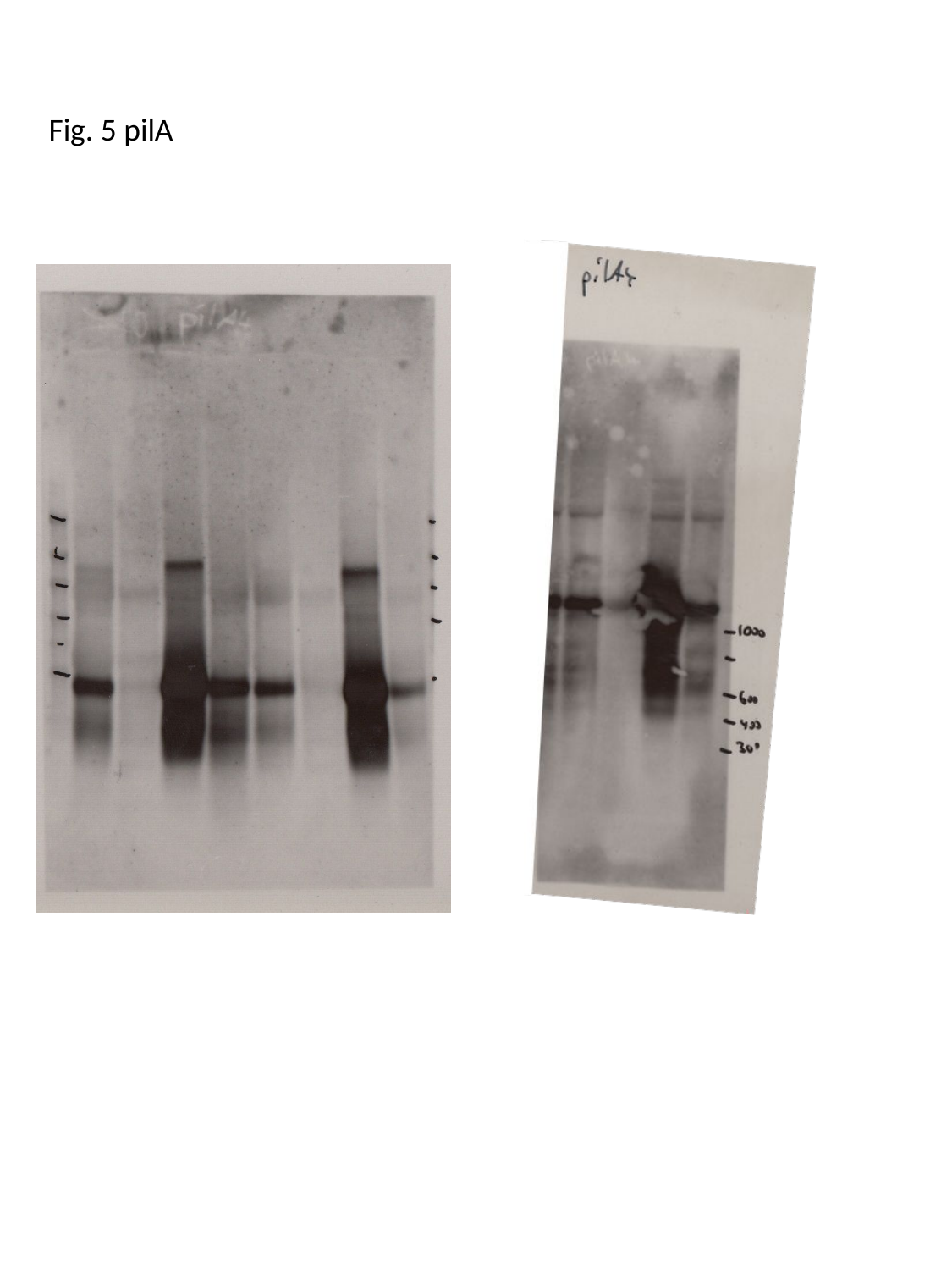

Fig. 5 pilA

## Slide 3
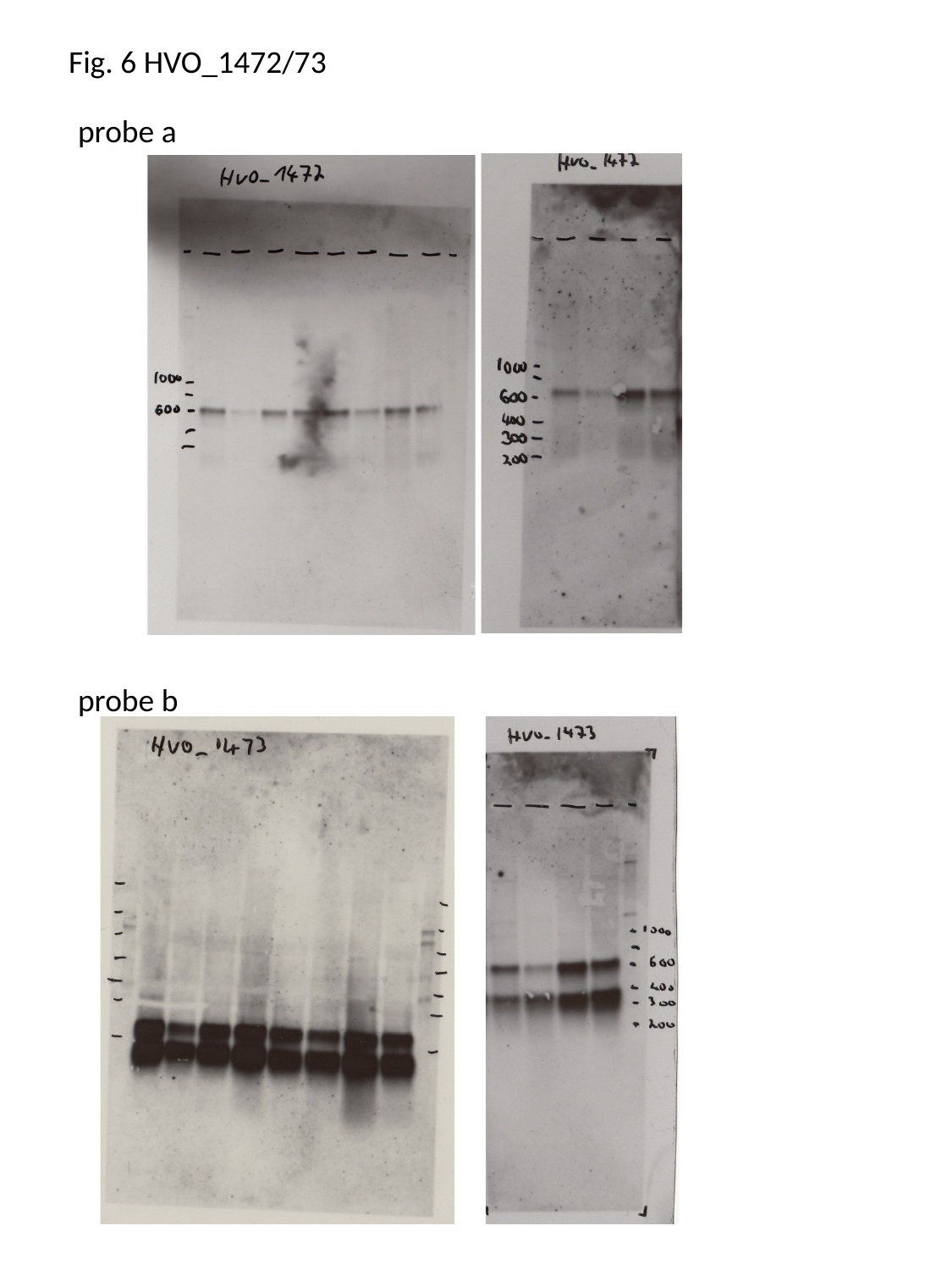

Fig. 6 HVO_1472/73
probe a
probe b

## Slide 4
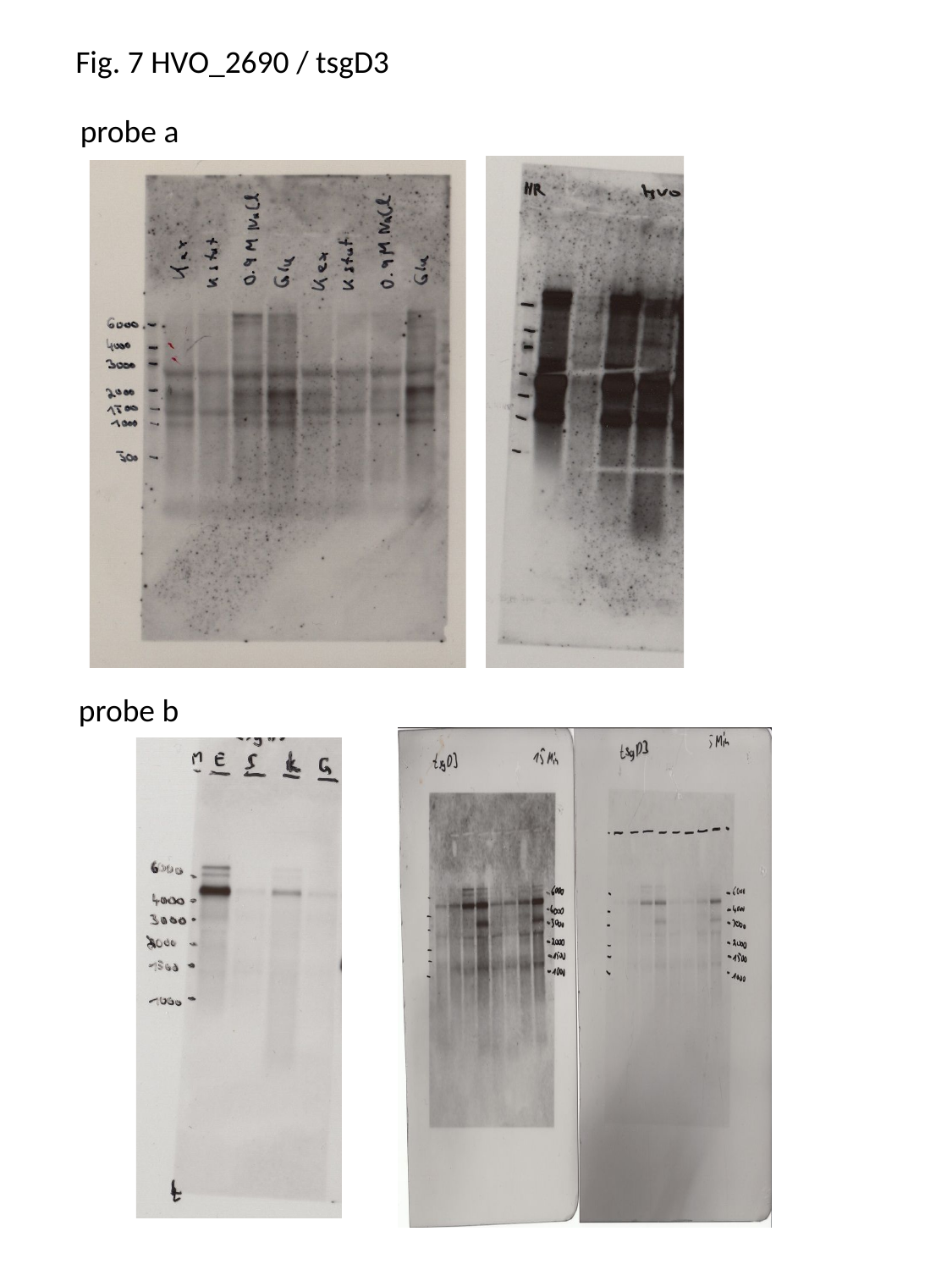

Fig. 7 HVO_2690 / tsgD3
probe a
probe b

## Slide 5
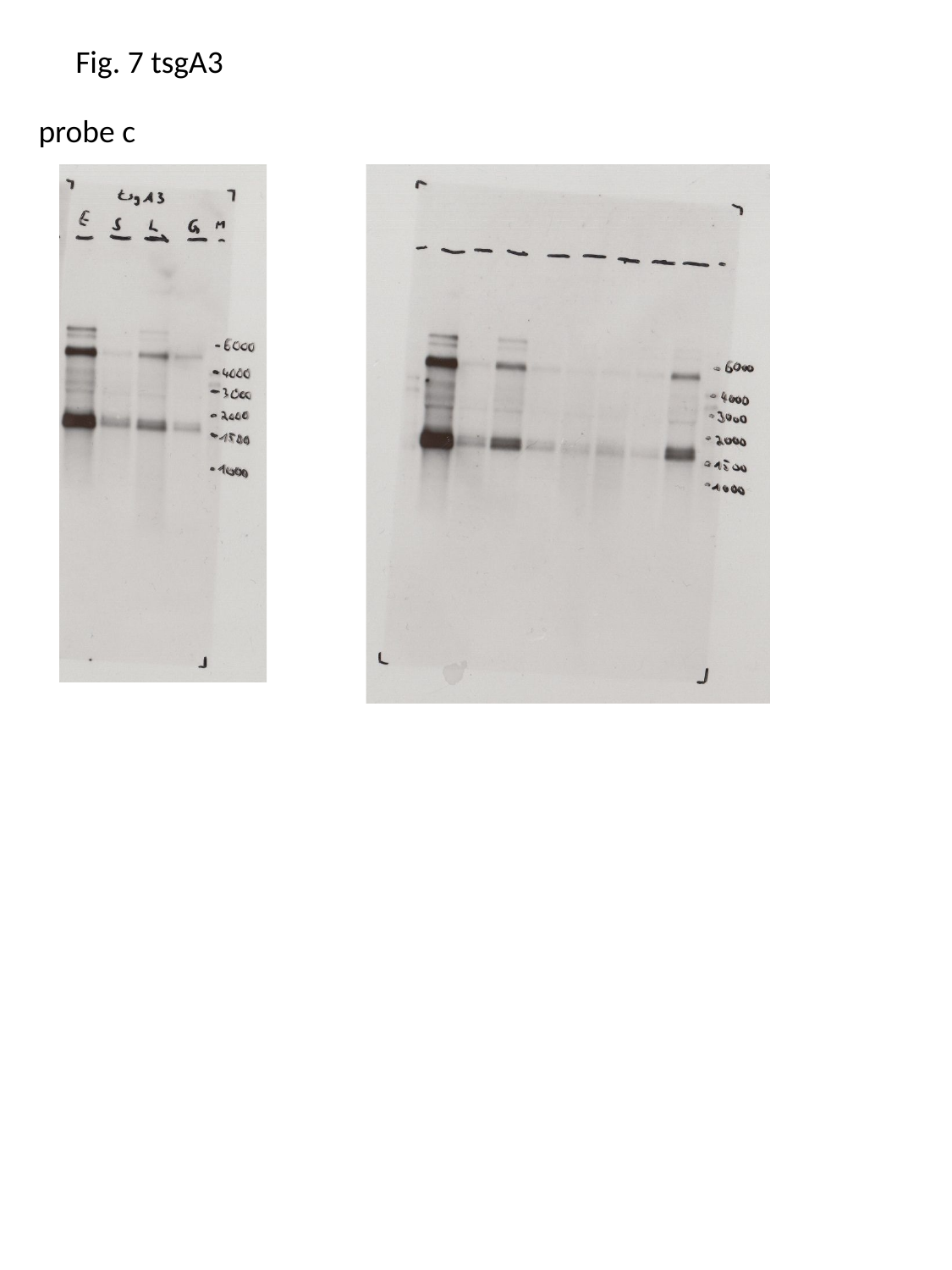

Fig. 7 tsgA3
probe c

## Slide 6
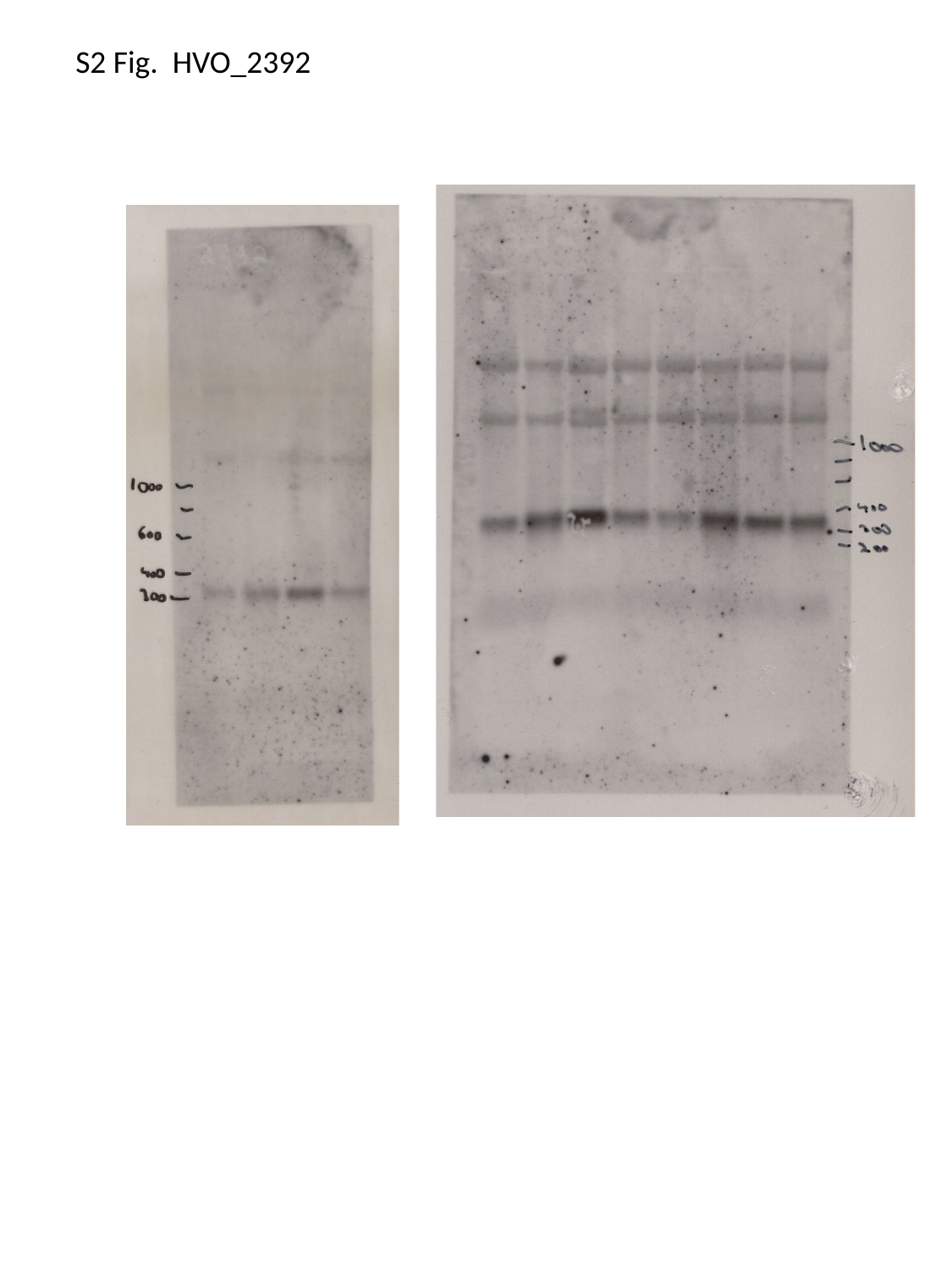

S2 Fig. HVO_2392

## Slide 7
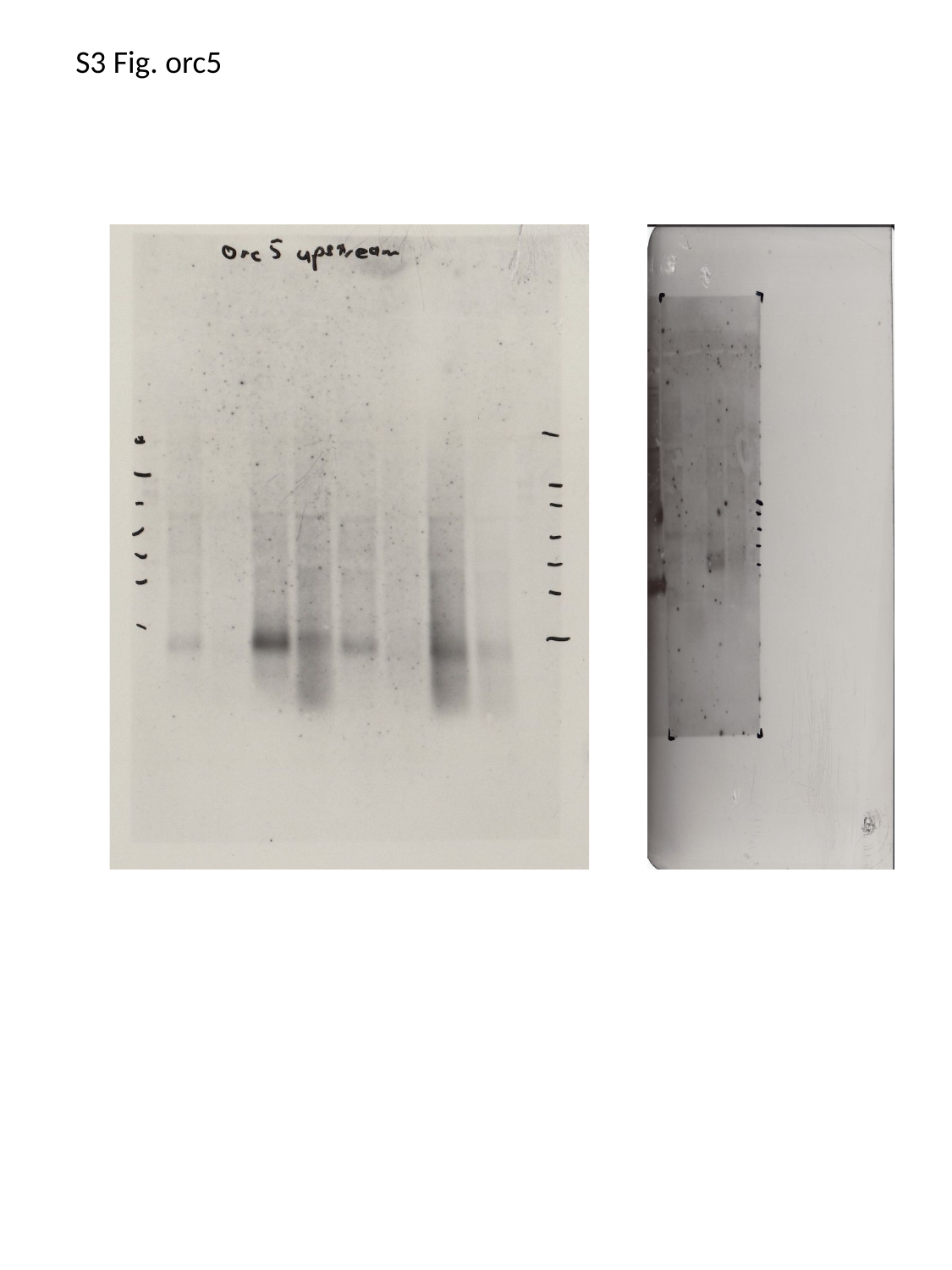

S3 Fig. orc5

## Slide 8
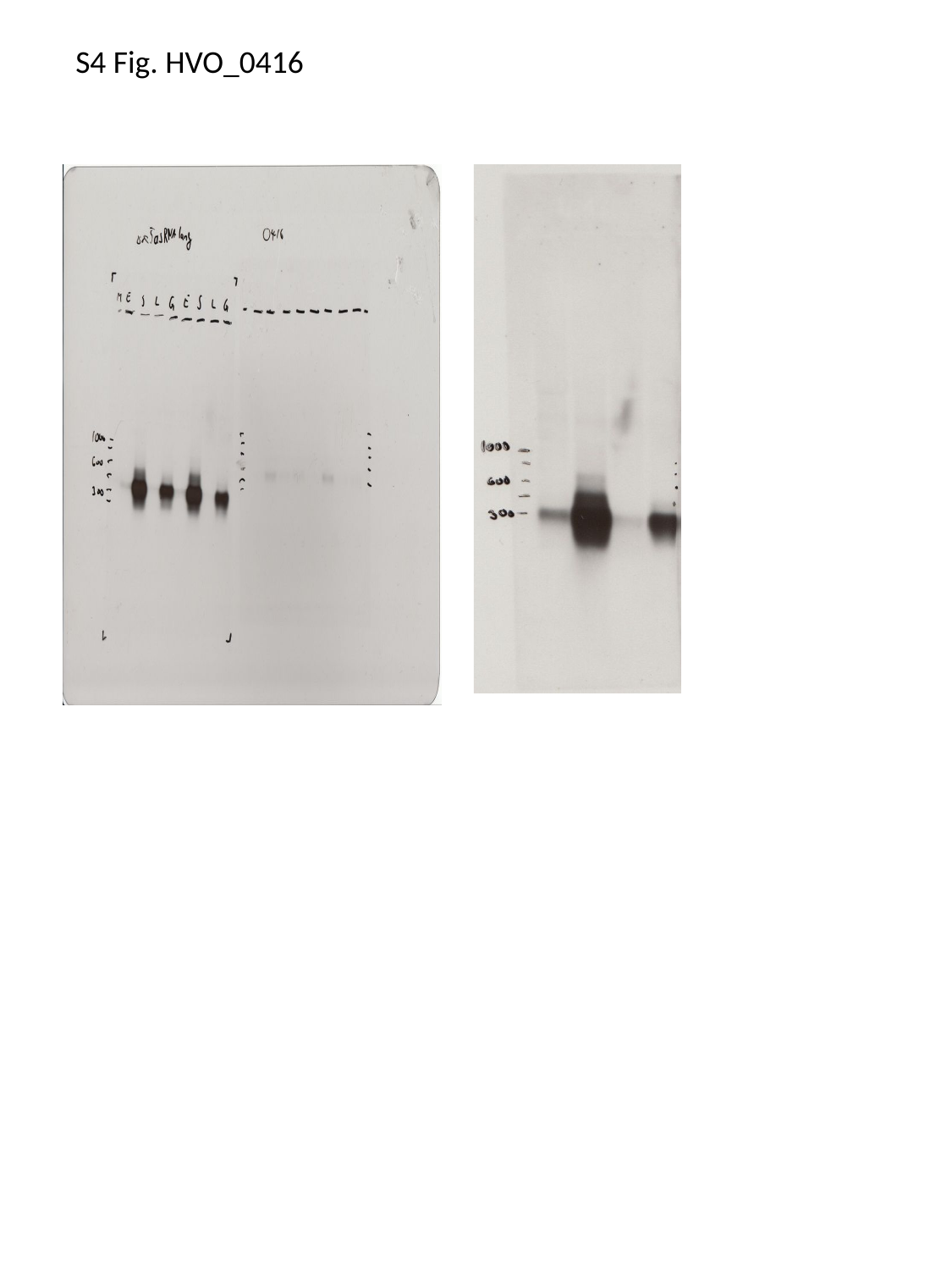

S4 Fig. HVO_0416

## Slide 9
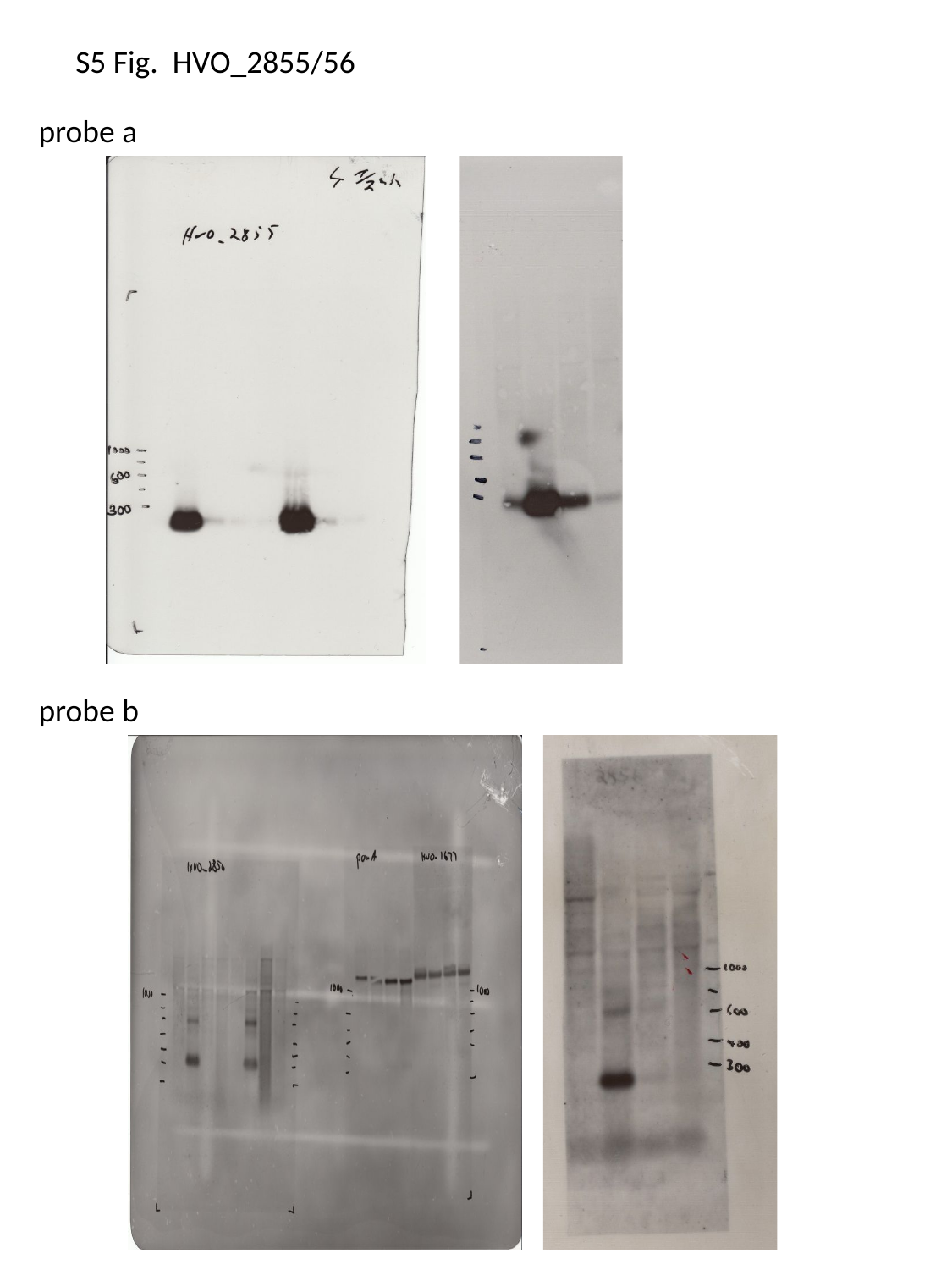

S5 Fig. HVO_2855/56
probe a
probe b

## Slide 10
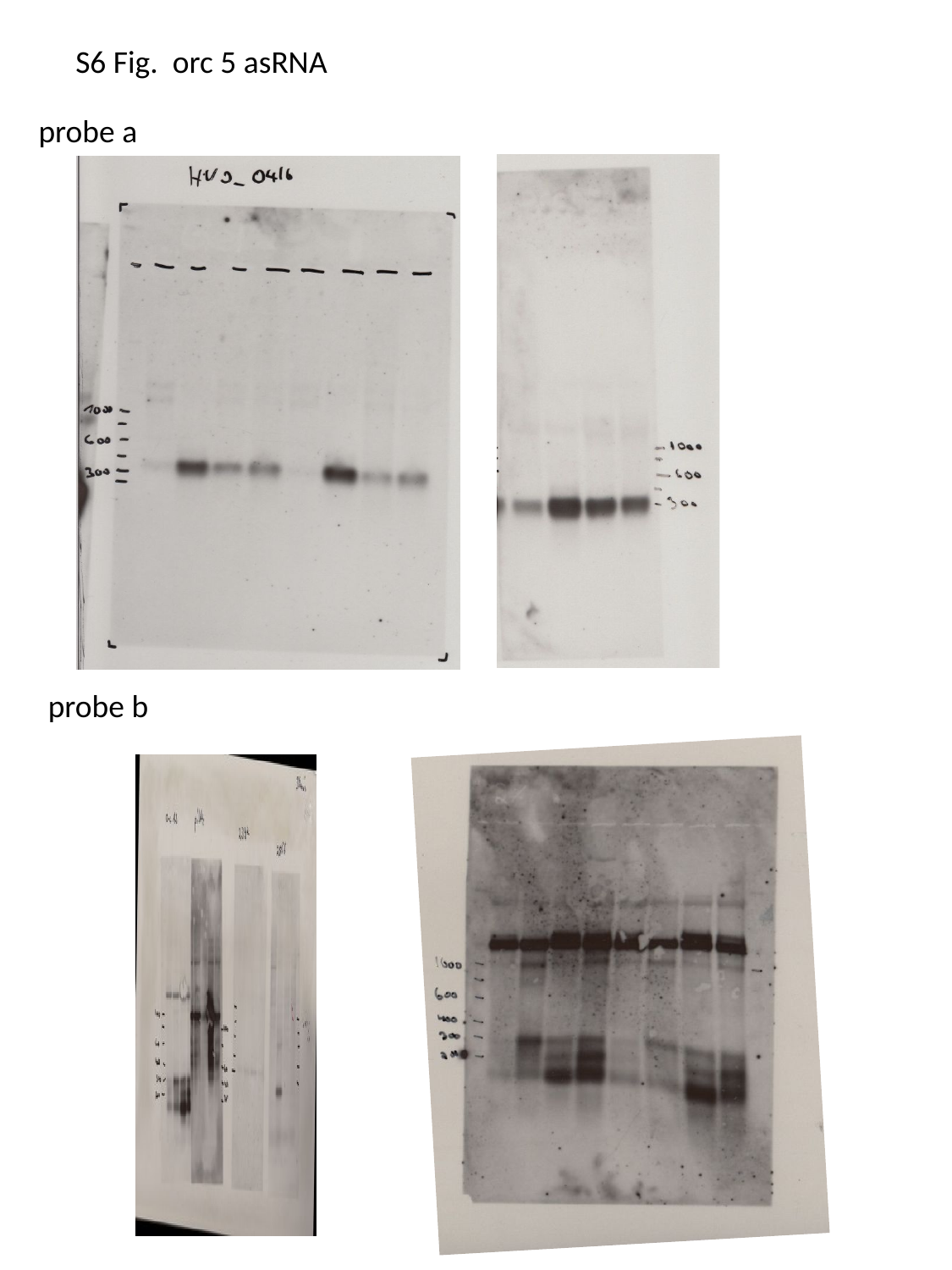

S6 Fig. orc 5 asRNA
probe a
probe b
